# Meiotic recombination dynamics in plants with repeat-based holocentromeres shed light on the primary drivers of crossover patterning

**DOI:** 10.1038/s41477-024-01625-y

**Published:** 2024-02-09

**Authors:** Marco Castellani, Meng Zhang, Gokilavani Thangavel, Yennifer Mata-Sucre, Thomas Lux, José A. Campoy, Magdalena Marek, Bruno Huettel, Hequan Sun, Klaus F. X. Mayer, Korbinian Schneeberger, André Marques

**Affiliations:** 1 Department of Chromosome Biology, Max Planck Institute for Plant Breeding Research, Cologne, Germany; 2 Laboratory of Plant Cytogenetics and Evolution, Department of Botany, Centre of Biosciences, Federal University of Pernambuco, Recife, Brazil; 3 Plant Genome and Systems Biology, German Research Centre for Environmental Health, Helmholtz Zentrum München, Neuherberg, Germany; 4 Max Planck Genome-Centre Cologne, Max Planck Institute for Plant Breeding Research, Cologne, Germany; 5 Faculty of Biology, Ludwig-Maximilians-Universität München, Planegg-Martinsried, Germany; 6 School of Life Sciences Weihenstephan, Technical University of Munich, Freising, Germany; 7 Cluster of Excellence on Plant Sciences (CEPLAS), Heinrich-Heine University, Düsseldorf, Germany; 8 Present Address: Department of Pomology, Estación Experimental de Aula Dei (EEAD), Consejo Superior de Investigaciones Científicas, Zaragoza, Spain; 9 Present Address: School of Automation Science and Engineering, Faculty of Electronic and Information Engineering, Xi’an Jiaotong University, Xi’an, China

**Keywords:** Genome evolution, Evolution, Cytogenetics, Next-generation sequencing, DNA methylation

## Abstract

Centromeres strongly affect (epi)genomic architecture and meiotic recombination dynamics, influencing the overall distribution and frequency of crossovers. Here we show how recombination is regulated and distributed in the holocentric plant *Rhynchospora breviuscula*, a species with diffused centromeres. Combining immunocytochemistry, chromatin analysis and high-throughput single-pollen sequencing, we discovered that crossover frequency is distally biased, in sharp contrast to the diffused distribution of hundreds of centromeric units and (epi)genomic features. Remarkably, we found that crossovers were abolished inside centromeric units but not in their proximity, indicating the absence of a canonical centromere effect. We further propose that telomere-led synapsis of homologues is the feature that best explains the observed recombination landscape. Our results hint at the primary influence of mechanistic features of meiotic pairing and synapsis rather than (epi)genomic features and centromere organization in determining the distally biased crossover distribution in *R. breviuscula*, whereas centromeres and (epi)genetic properties only affect crossover positioning locally.

## Abstract (web summary)

Castellani, Zhang and colleagues found that centromeres and (epi)genetic features influence local crossover positions during meiotic recombination in a plant with diffused centromeres, whereas chromosome synapsis dynamics seems key to broad-scale crossover patterning.

## Main

During meiosis, homologous chromosomes (homologues) undergo meiotic recombination, exchanging genomic material between them. This exchange is initiated by physiologically induced DNA double-strand breaks (DSBs)^1,2^. The formation of meiotic DSBs is commonly resolved via crossovers (COs) or other recombination outcomes, called non-COs^3^. COs can be divided into two classes^4,5^. In plants, class I COs are the most prevalent and are sensitive to interference—they do not occur near each other along a chromosome—whereas class II COs are insensitive to interference and can accommodate around 10% of the total COs^4^. Although the repertoire of meiotic-specific proteins is largely conserved across eukaryotes^6,7^, species-specific adaptations can occur^8^.

The distribution of COs is typically associated with the distribution of genetic and epigenetic ((epi)genetic) features^9,10^. In most eukaryotes, the CO frequency correlates positively with gene/euchromatin density^11,12^, but is lower in heterochromatic regions, including (peri)centromeres^13,14^. In monocentric species, centromeres are single-defined structural entities and are typically repeat-based^15^. Recombination is largely suppressed at and in the proximity of centromeres in these species, a phenomenon called the centromere effect^16–19^. This feature, combined with (epi)genetic elements, is believed to cause a distal bias in CO frequencies^9,12,20–23^. However, how these factors influence meiotic recombination patterning at broad and local scales is still elusive.

Monocentricity is not the only centromeric organization adopted by eukaryotes. For instance, holocentric species harbour multiple centromeric determinants over the entire length of their chromosomes^24,25^. Holocentricity has evolved independently multiple times in nematodes, insects and plants^26,27^. In the holocentric animal models *Caenorhabditis elegans* and silk moth (*Bombyx mori*), holocentromeres do not associate with a specific sequence and have variable dynamics^28,29^. By contrast, holocentric plants of the genus *Rhynchospora* (beaksedges) display specific repeats constitutively loaded with holocentromeres in both mitosis and meiosis^30,31^. Recently, we sequenced the genomes of three beaksedges (*R. breviuscula*, *R. pubera* and *R. tenuis*) and determined that each chromosome harbours hundreds of short arrays (~20 kb each) of the specific Tyba (meaning “abundance” in Tupi-Guarani, a language spoken by many Brazilian native tribes) tandem repeat that are distributed genome-wide and specifically associated with centromeric histone H3 protein (CENH3)^32^. This particular chromosome organization is associated with a remarkably uniform distribution of genes, repeats and (epi)genetic features, in contrast to the compartmentalized chromosome organization found in many monocentric eukaryotes^32^. Remarkably, each individual centromeric unit in *R. pubera* showed epigenetic regulation similar to that in other plant monocentromeres^23,32^. Moreover, meiosis has been only studied in holocentric plants regarding their intriguing ‘inverted meiosis’ and centromere organization^26,33–35^. Thus, *Rhynchospora* offers an excellent model to study the mechanisms of CO formation in the absence of the effect of the monocentromere, while sharing similar centromere chromatin (epi)genetic properties.

Here, we use *R. breviuscula* as a model to study meiotic recombination dynamics in the absence of both a localized centromere and a compartmentalized chromosome organization, features that potentially mask underlying factors affecting CO distribution in most genomes. Using a combination of immunocytochemistry, chromatin and DNA analysis, and CO calling from single-gamete sequencing, we offer an overview of meiotic recombination dynamics and distribution for a species with repeat-based holocentromeres. Remarkably, the megabase-scale CO distribution did not correlate with any (epi)genomic feature analysed. We show that the CO distal bias is achieved even in the absence of both a monocentromere and a correlation with (epi)genomic features. We found that COs are suppressed inside repeat-based centromeric units but not in their proximity, indicating the absence of a centromere effect. Moreover, our cytological data suggest that synapsis dynamics starting from chromosomal ends influences the broad-scale recombination landscape in *R. breviuscula*. We propose that centromere and (epi)genetic features play a role in CO positioning, but only at the fine scale.

## Results

### Canonical dynamics of early meiosis I in *R. breviuscula*

**Extended Data Fig. 1:**
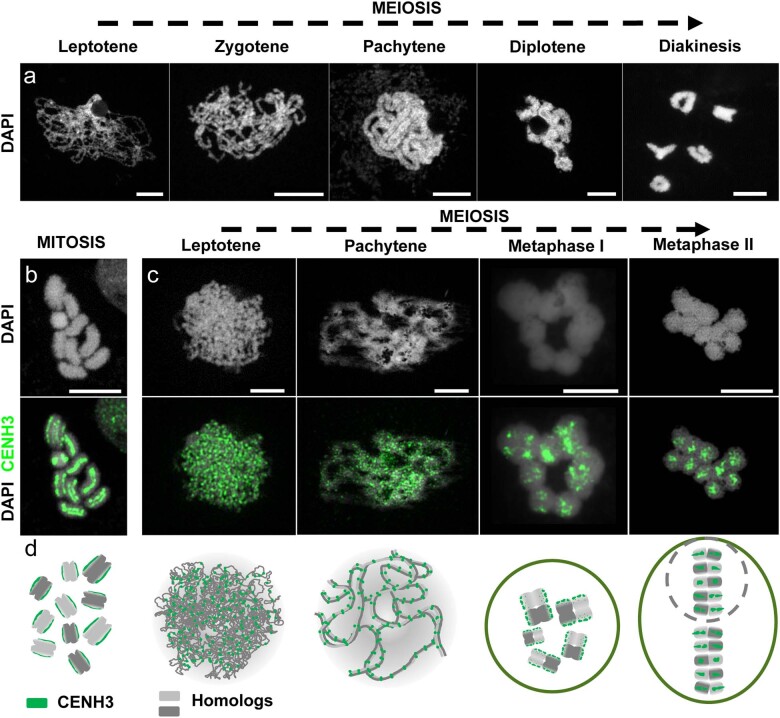
Chromosome spreads and immunolocalisation in male *R. breviuscul*a meiocytes. **(a)** Meiotic stages are displayed, including leptotene, zygotene, pachytene, diplotene and diakinesis. DAPI staining was consistent in all cells analysed in our immunocytochemistry experiments (see main text). **(b–c)** Immunolocalisation was performed against the centromeric protein CENH3, which appears as lines during mitosis **(b)** and as clusters during meiosis **(c)**. A maximum projection is shown, and the DNA was counterstained with DAPI. **(d)** Model of differential mitotic and meiotic holocentromere organization in the holocentric plant *R. breviuscula*. CENH3 behaviour was consistent in all cells (n = 31) in 4 independent experiments. Scale bars, 5 µm.

Chromosome spreads on male meiocytes of *R. breviuscula* revealed all the classical prophase I stages with the occurrence of five bivalents, indicating the formation of at least one CO per homologue pair (Extended Data Fig. 1a). Moreover, we confirmed the holocentric nature of *R. breviuscula* chromosomes by showing the localization of CENH3 in mitosis and meiosis. Similar to what has been reported in *R. pubera*^31^, CENH3 appears as lines during mitotic metaphase, but undergoes restructuring into more irregular clusters during meiosis (Extended Data Fig. 1b–d).

**Fig. 1:**
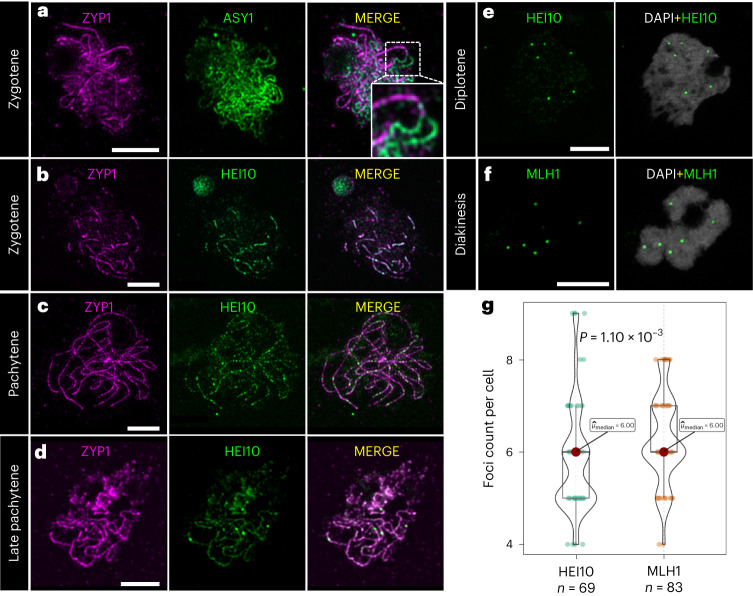
Immunolocalization of ZYP1, ASY1, HEI10 and MLH1 in prophase I. **a**, In zygotene, synapsis is visualized as the loading of ZYP1 while the ASY1 signal disappears. The insert shows a magnification of two unpaired chromosomes, represented by ASY1, coming together to synapse, with loss of the ASY1 signal and the loading of ZYP1. The behaviour of ASY1 + ZYP1 was consistent in all cells at zygotene (*n* = 16) in nine independent experiments. **b**, In early zygotene when synapsis starts, HEI10 is immediately loaded as many closely spaced foci, forming an irregular and patchy signal and co-localizing with ZYP1. **c**, In pachytene, HEI10 is visible as lines that co-localize with ZYP1. **d**, In late pachytene, the linear signal of HEI10 co-localizes with ZYP1 but becomes weaker, except for a few highly intense foci. The behaviour of HEI10 + ZYP1 was consistent in all cells from zygotene to late pachytene (*n* = 61) in ten independent experiments. **e**, During the diplotene and diakinesis stages, HEI10 appears as foci only on bivalents, with no linear signal. The behaviour of HEI10 from diplotene to diakinesis was consistent in all cells analysed (*n* = 69) in ten independent experiments. **f**, MLH1 appears in diplotene and diakinesis as foci on bivalents, representing chiasmata. The behaviour of MLH1 from diplotene to diakinesis was consistent in all cells analysed (*n* = 83) in eight independent experiments. **g**, Box plots of HEI10 and MLH1 foci count at late prophase I. Two-sided Mann–Whitney *U*-tests were performed. The box plots are comprised of minima, first quartile (Q1), median ($\hat{\mu}_{\mathrm{median}}$; HEI10 = 6.00, MLH1 = 6.00), third quartile (Q3) and maxima following the definition of ggplot2 in R. Being produced in the same animal, co-localization of these two markers could not be performed. Maximum projections are shown for microscope images, with chromosomes counterstained with DAPI. Scale bar, 5 µm.

**Extended Data Fig. 2:**
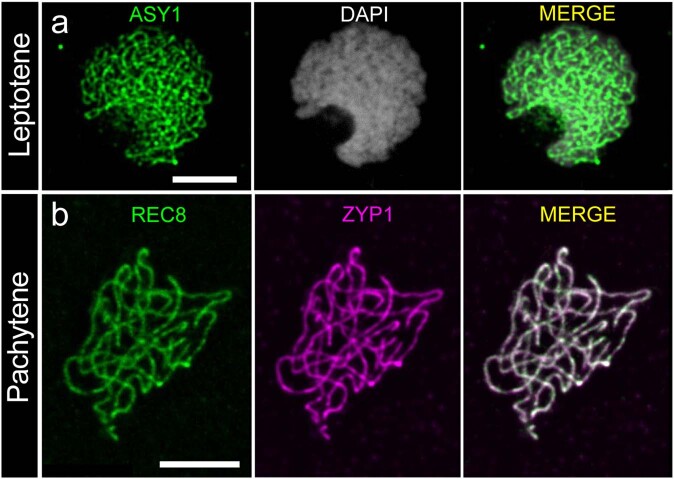
Immunolocalisation of ASY1, REC8, and ZYP1 from leptotene to pachytene of meiotic prophase I. **(a)** ASY1 (green) appears as a linear signal on unpaired chromosomes. Behaviour of ASY1 was consistent in all meiocytes analysed (n = 10) among 6 independent experiments. **(b)** Full co-localisation of cohesin protein REC8 (green) and ZYP1 (magenta) at pachytene. Behaviour of REC8+ZYP1 was consistent in all meiocytes analysed (n = 12) among 3 independent experiments. A maximum projection is shown; chromosomes were counterstained with DAPI. Scale bars, 5 µm.

We then investigated ASY1 (refs. ^36,37^) and ZYP1 (refs. ^38–42^) as indicators of conserved and functional axis formation and synapsis, respectively. The ASY1 signal was present along the entire length of unsynapsed chromosomes at leptotene (Extended Data Fig. 2a). During zygotene, paired ASY1 linear signals could be followed until they converged and lost intensity (Fig. 1a), whereas ZYP1 was gradually loaded onto synapsed chromosomes (Fig. 1a,b). In pachytene, complete synapsis was evidenced by the linear ZYP1 signal along the full length of chromosomes (Fig. 1c). We also tested whether the meiosis-specific alpha-kleisin REC8, a protein responsible for sister chromatid cohesion^43^, is conserved in *R. breviuscula*. We detected a conserved linear REC8 signal at pachytene co-localizing with ZYP1, forming a linear signal along the entirely synapsed chromosome (Extended Data Fig. 2b). Thus, pairing, cohesion and synapsis are conserved in the holocentric plant *R. breviuscula*, resembling those in monocentric models.

We further studied the behaviour of HEI10, a RING-family E3 ligase that has been characterized in plants, fungi and animals^44–47^. HEI10 has been proposed to interact with both early and late recombination proteins, stabilizing recombination intermediates and advancing them into class I COs^44,46–50^. In *R. breviuscula*, when synapsis started in early zygotene, HEI10 was immediately loaded as a dotty linear signal co-localizing with the first ZYP1 signals (Fig. 1b). At early pachytene, HEI10 progressed to form an increasingly linear signal co-localizing with ZYP1 along the entirety of synapsed chromosomes (Fig. 1c). Throughout pachytene the HEI10 signal became discontinuous along chromosomes, and a few foci increased in intensity (Fig. 1d). We think that these are putative class I CO sites. In diplotene and diakinesis, only high-intensity foci remained (Fig. 1e and Supplementary Fig. 1). Thus, the dynamic behaviour of HEI10 is conserved in *R. breviuscula*, displaying the ‘coarsening’ dynamics recently proposed in *Arabidopsis*^51–53^.

Another established marker for meiotic recombination is the mismatch repair protein MLH1 (MUTL-HOMOLOG 1), which has a meiosis-specific resolvase activity to process double Holliday junctions into final class I COs. MLH1 interacts with MSH4 and MSH5, specifically marking class I COs^54–56^. In *R. breviuscula*, the MLH1 signal appears exactly at diplotene, after synaptonemal complex (SC) disassembly, and persists as bright foci until the end of diakinesis (Fig. 1f and Supplementary Fig. 2). We observed a median number of six foci for both HEI10 (*n* = 69) and MHL1 (*n* = 83) (Fig. 1g), which is consistent with the formation of at least one CO per homologue pair. Foci counting of these two markers showed a small but significant difference (Fig. 1g). We also observed at least one homologue pair with more than one CO; that is, ring bivalent (Supplementary Fig. 3). Thus, in contrast to the holocentric *C. elegans* where strictly only one CO is allowed per bivalent^57^, *R. breviuscula* can form more than one CO per bivalent. Furthermore, our results confirm a canonical and conserved early meiosis programme in *R. breviuscula*.

### Pollen single-cell sequencing allows genome-wide CO detection

**Extended Data Fig. 3:**
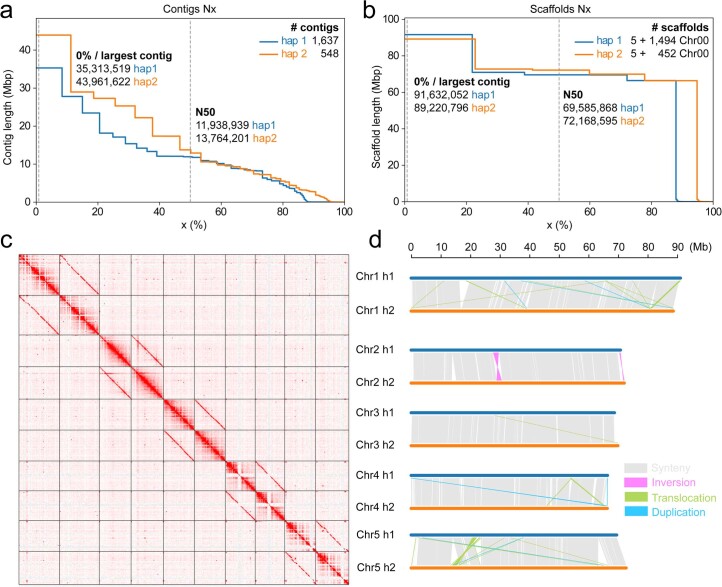
Phasing and structural variation of the *R. breviuscula* heterozygous genome. **(a–b)** Assembly statistics of the phased contigs **(a)** and scaffolds **(b)** for haplotype 1 and haplotype 2. **(c)** Hi-C scaffolding of the five haplotype-phased pseudochromosomes. Homozygous regions between the haplotypes are seen as clear regions depleted of signals on the Hi-C map. **(d)** Synteny blocks and structural variants (>10 kb) identified between the two haploid assemblies. Note the overall high synteny between the two haplotypes. Synteny blocks were computed with SyRI and plotted with plotsr.

We set out to determine whether meiotic recombination in *R. breviuscula* is affected by the genome-wide distribution of holocentromeres. To identify COs from a single *R. breviuscula* individual, we took advantage of its 1% heterozygous genome^32^ to generate a fully haplotype-phased chromosome-scale reference genome (Extended Data Fig. 3, Supplementary Tables 1 and 2 and Methods). Moreover, *R. breviuscula* is an outbred species with high levels of self-incompatibility, which hampers the standard detection of COs, typically involving the time-consuming generation of segregating offspring. Thus, we obtained only 63 F_1_ plants by manual selfing-pollination that were sequenced at threefold genome coverage. Although, we detected 378 CO events at a very high resolution (CO resolution: median 334 bp, mean ~2 kb), the limited number of detected COs did not allow comprehensive drawing of the recombination landscape (Supplementary Fig. 4).

**Fig. 2:**
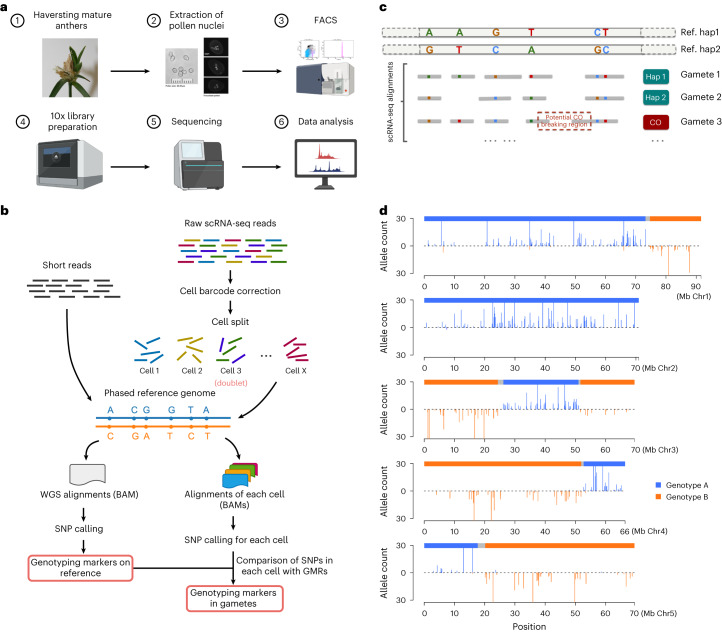
Overview of CO calling by adapting scRNA-seq to *R. breviuscula* gametes. **a**, Pollen sampling, library preparation and scRNA-seq pipeline. FACS, fluorescence activated cell sorting. **b**, Diagram of the strategy used for identifying genotyping markers on the reference genome by mapping short reads, and markers in gametes by mapping scRNA-seq reads, across a large number of gametes to the reference genome. GMR, genotyping marker on reference genome; WGS, whole-genome sequencing. **c**, Diagram of the identification of potential CO events after the alignment of scRNA reads from each gamete to the phased reference genome. **d**, An example of genotype definition by markers in a real pollen nucleus with the cell barcode AAGACTCTCATCCTAT.

Next, we aimed to provide a shortcut for constructing a robust broad-scale CO landscape for *R. breviuscula*. Gametes carry the outcome of meiotic recombination and can be obtained in large numbers in a relatively inexpensive manner from pollen grains. Thus, by adapting a strategy based on the gamete-binning method described by Campoy et al.^58^, we identified genome-wide CO events by conducting 10x Genomics single-cell RNA sequencing (scRNA-seq) on extracted nuclei from pollen grains (male gametes) of *R. breviuscula* (Fig. 2a and Methods), with the caveat that pollen nuclei show a relatively low abundance of transcripts (Supplementary Fig. 5), limiting the resolution of CO detection.

To genotype the haploid gamete genomes, we obtained genome-wide haplotype-specific markers by aligning ~26 Gb Illumina whole-genome short reads to the phased haplotype 1 of *R. breviuscula*. We detected 820,601 haplotype-specific single nucleotide polymorphisms (SNPs; ~1 SNP per 449 bp) and used them as markers for genotyping (Fig. 2b and Supplementary Figs. 6 and 8a).

We pre-processed scRNA sequences by correcting barcodes, demultiplexing, and removing doublets and cells with a low number of reads (Supplementary Fig. 7 and Methods), resulting in a final set of 1,641 pollen nuclei with at least 400 markers (~1 marker per Mb). These markers covered almost the entire length of all five chromosomes (Supplementary Fig. 8b), ensuring genome-wide CO detection (CO resolution: median ~1.5 Mb, mean ~2.24 Mb). We detected 4,047 COs in the 1,641 pollen nuclei by inspecting genotype conversions, as indicated in Fig. 2c,d. Overall, we delineated a complete and detailed pipeline to detect COs in an economical way using high-throughput scRNA-seq of gametes from a single heterozygous individual (Fig. 2).

### Deciphering the CO landscape in a holocentric plant

**Fig. 3:**
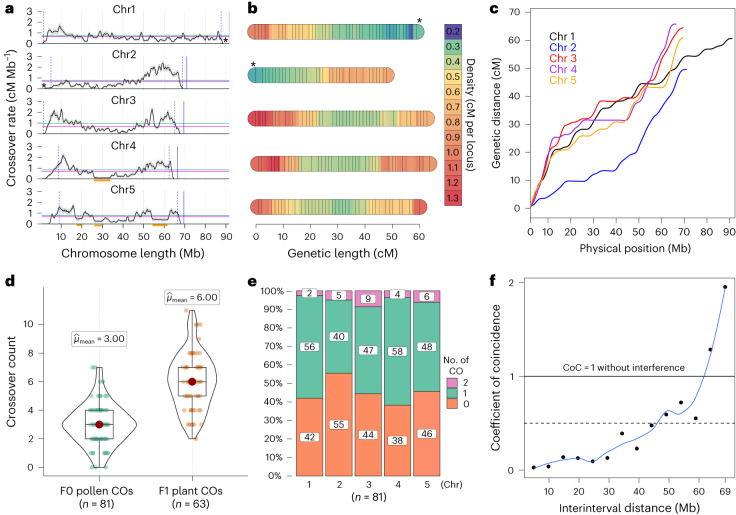
Gamete-sequencing derived meiotic recombination dynamics in *R. breviuscula*. **a**, Recombination landscape of the five chromosomes in *R. breviuscula* achieved by computing 4,047 COs from 1,641 pollen nuclei. The black line shows the CO rate; shadow ribbons indicate one standard deviation from mean CO rates; blue dashed vertical lines indicate the start and end of confident CO rate computation (Supplementary Fig. 9); blue solid vertical lines mark the chromosomal end; magenta horizontal lines indicate the genome-wide average CO rate; green horizontal lines are the chromosome-wide average CO rate; and orange bars indicate large (>2 Mb) homozygous regions with a reduced number of markers. **b**, Genetic linkage map computed from COs in 81 F_0_ pollen nuclei having more than 2,000 markers. Genetic length density is indicated by the coloured scale. A set of 705 markers was selected using a 500 kb sliding window through all markers defined against the reference (Methods). The terminal locations of the *35S rDNA* locus on chr1 and chr2 are indicated by asterisks in **a** and **b**. **c**, Marey map calculated from the linkage map in **b**. Marey maps for each chromosome (colour lines) show genetic position as a function of physical position. **d**, CO number derived by counting CO events from the genetic analysis in pollen nuclei having more than 2,000 markers compared with the one extrapolated from the F_1_ offspring. The box plots are comprised of minima, Q1, mean, Q3 and maxima following the definition of ggplot2 in R. N indicates biologically independent pollen nuclei and F_1_ offspring individuals used for CO detection. **e**, Distribution of CO number for each single chromatid in gametes. Note the higher incidence of double COs on chr3. **f**, CoC curve in pollen nuclei (*n* = 1,641). Chromosomes were divided into 15 intervals, with random sampling at CO intervals, to calculate the mean CoC of each pair of intervals.

Counting the occurrence of COs in chromosome-wide genomic intervals across 1,641 pollen nuclei, we computed the CO rates along chromosomes and established a recombination map for *R. breviuscula* with known repeat-based holocentromeres (Fig. 3a and Supplementary Fig. 9). Unexpectedly, we observed an apparent distal bias in CO distribution, whereas centre regions maintain rather low CO rates, mostly lower than the mean CO rate genome-wide. Remarkably, chromosome 3 (chr3), chr4 and chr5 showed CO distal bias at both ends (Fig. 3a). By contrast, chr1 and chr2 showed a prominent increase in CO rate at only one chromosomal end (CO rate above the genome-wide mean), whereas the other end showed comparatively low CO rates similar to centred regions. Interestingly, these low recombination ends were correlated with the localization of 35S ribosomal DNA loci (nucleolar organizing regions) (Fig. 3a,b). We thus revealed an uneven distribution of CO rates at the megabase-scale, despite the presence of holocentric chromosomes in *R. breviuscula*.

**Extended Data Fig. 4:**
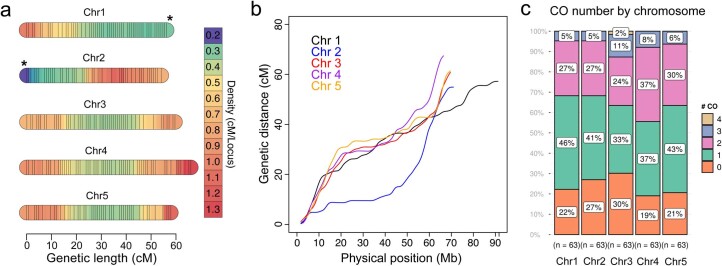
Recombination frequencies in the F1 recombinant offspring of *R. breviuscula*. **(a)** Genetic linkage map using 378 COs detected in our 63 F1 individuals with density indicated by colouring. The 705 markers were selected using a 500-kb sliding window through all markers defined on the reference (**see Methods**). **(b)** Marey map calculated from the linkage map in **a**. Marey maps for each chromosome (colour lines) show genetic position as a function of physical position. **(c)** Distribution of CO numbers individual chromosomes in the 63 F_1_ recombinant individuals.

We further compared the number of genetically identified CO events derived from pollen nuclei with more than 2,000 markers (*n* = 81; 243 COs) and F_1_ selfed offspring data. Although, the computed total linkage map length obtained from both pollen and F_1_ offspring was 300 cM, slight differences in genetic length were observed among the chromosomes (Fig. 3b,c and Extended Data Fig. 4a,b). Differences in genetic length for the same chromosome between pollen and F_1_ could be due to the occurrence of heterochiasmy^40^, a phenomenon that causes strong differences in recombination rates between female and male meiosis. Remarkably, chr3, chr4 and chr5 had longer genetic lengths among all *R. breviuscula* chromosomes (Fig. 3b,c and Extended Data Fig. 4a,b). This is evident considering these three chromosomes have distal CO bias at both ends compared with only one in chr1 and chr2.

On average, we detected around three COs per haploid gamete, or 0.6 CO per chromatid (Fig. 3d,e), which is approximately half of the COs detected in our segregating offspring and HEI10/MLH1 foci (Figs. 1g and 3d), because gametes contain only one chromatid of each recombined chromosome. Furthermore, all single chromatids had, on average, one CO in half of these gametes (*n* = 81), whereas double COs appeared in approximately 5% of the 81 gametes considered (Fig. 3e). Chromosome 3 showed the highest frequency of double COs (9%; Fig. 3e and Extended Data Fig. 4c). Although we cannot discard the occurrence of class II COs, our results suggest that most COs formed in *R. breviuscula* are of class I.

We also tested whether CO interference occurred in *R. breviuscula*. A chi-squared goodness-of-fit test suggested that the observed COs did not reside randomly, and a further dispersion test revealed CO number distribution was underdispersed because of the effect of CO interference (Supplementary Fig. 10a). We also computed the coefficient of coincidence (CoC) for COs across the genome. The CoC measures the observed frequency of double COs over the expected frequency (Methods). The CoC curve of all chromosomes showed that the coefficients are below 1 for genomic intervals with distances of less than around 60 Mb (Fig. 3f and Supplementary Fig. 10b), meaning that the frequency of double COs is lower than expected. This result indicates the presence of strong CO interference in *R. breviuscula*.

### CO landscape is independent of (epi)genomic features

**Fig. 4:**
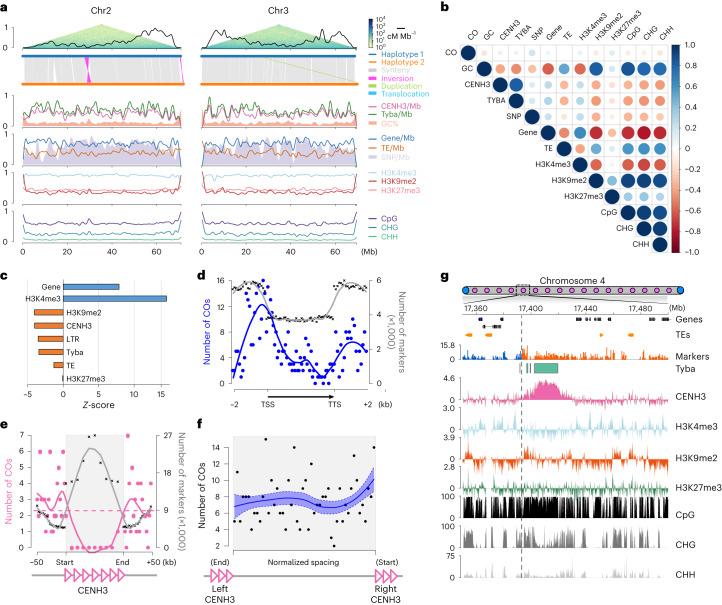
Broad- and fine-scale correlations between CO patterning and (epi)genomic features in *R. breviuscula*. **a**, Chromosome distribution of the CO rate coupled with different (epi)genetic features. (Upper) Recombination landscape (black line) created with COs detected in all single-pollen nuclei (*n* = 1,641), coupled with Omni-C data. Synteny analysis and detected structural variants between the two haplotypes. For the *y* axes, all features were scaled [0, 1], with 1 indicating a maximum of 2.34 for CO frequency (cM Mb^−1^), 5 for Tyba array density, 6 for CENH3 domain density, 7,205 for SNP density, 88 for gene density and 227 for TE density. GC [33.3, 46.6], H3K4me3 [–1.494, 0.231], H3K9me2 [–1.20, 1.84] and H3K27me3 [–0.671, 0.491] are scaled to [0, 1] by their minima and maxima. Methylated CG (mCG), methylated CHG (mCHG) and CHH are original values (0–100%). All features were smoothed using a 1 Mbp sliding window and 250 kbp step size. **b**, Correlation matrix illustrating the correlation coefficients of 378 high-resolution COs detected in 63 selfed F_1_ offspring with all available (epi)genomic features. Blue, positive correlations; red, negative correlations. Colour intensity and the size of the circle are proportional to the correlation coefficients. Pearson correlation coefficients for each pair of all features under a 1 Mb smoothing window and 250 kb step size. **c**, *Z*-score of the overlapped CO numbers with different (epi)genetic features. **d**, CO frequency (blue line) at genic regions. TSS, transcription start site; TTS, transcription termination site; grey line, marker density. **e**, Fine-scale CO frequency at CENH3 domains. The dashed horizontal line is the genome-wide mean CO count within a 2.5 kb interval. **f**, Random distribution of the relative distance of CO positions to the end of the left and to the start of the right CENH3 domain. The solid blue line was predicted by local polynomial regression fitting (loess function from R) using data from 378 COs from our F_1_ offspring. The dashed blue band shows a range of one standard error above and below the fitted line. Pink-bordered triangles schematically represent CENH3 domains. **g**, Magnified view of one of the five COs placed closed to a region containing CENH3-positive chromatin and Tyba repeats. The CO resolution in this case is 200 bp. CO is indicated by the grey dashed line showing the haplotype switch (blue to orange) in the marker density track.

**Extended Data Fig. 5:**
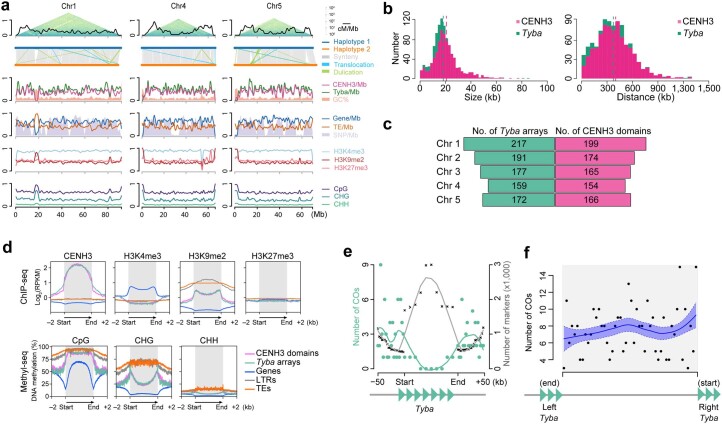
Epigenetic regulation of repeat-based holocentromeres and fine-scale correlation of CO positions with *Tyba* repeats. **(a)** Chromosome distribution of the CO rate coupled with different (epi)genetic features. Top: recombination landscape (black line) created with COs detected in all single-pollen nuclei (n = 1,641), coupled with Omni-C chromosome conformation capture contacts. Synteny analysis and detected the structural variants between the two haplotypes. For the y-axes, all features were scaled [0,1], with 1 indicating a maximum of 2.34 for recombination frequency (cM/Mb), 5 for *Tyba* density, 6 for CENH3 density, 7205 for SNP density, 88 for gene density, and 227 for TE density. GC [33.3, 46.6], H3K4me3 [–1.494, 0.231], H3K9me2 [–1.20, 1.84], and H3K27me3 [–0.671, 0.491] are scaled to [0,1] by their minima and maxima. mCG, mCHG and CHH are original values (0 to 100%). All features were smoothed by 1Mbp sliding window and 250kbp step size. **(b)** Size (left) and spacing (right) length distribution of CENH3 domains and *Tyba* arrays. CENH3 domain median size is 19,156 bp and the mean size is 20,697 bp. The median of *Tyba* array size is 17,424 bp and the mean is 18,220 bp. CENH3 domain median spacing is 378,467 bp and the mean is 401,763 bp. The median of *Tyba* array spacing is 354,850 bp and the mean is 374,310 bp. **(c)** Number of *Tyba* arrays (left) and CENH3 domains (right) for each chromosome annotated in the reference haplotype genome. **(d)** Enrichment of CENH3, H3K4me3, H3K9me2, and DNA methylation in the CpG, CHG, and CHH contexts from the start and end of different types of sequences: CENH3 domains, *Tyba* repeats, genes, LTRs, and TEs. ChIP-seq signals are shown as log2 (normalised RPKM ChIP/input). Grey boxes highlight the modification enrichment over the body of each sequence type. **(e)** CO frequency within *Tyba* arrays. **(f)** Random distribution of the relative distance of CO positions to the end of the left and to the start of the right *Tyba* array. The median of CO resolution is 334 bp and the mean is about 2 kb. The solid blue line was predicted by Local Polynomial Regression Fitting (loess function from R) using data from 63 F_1_ recombinant offspring and a total of 378 COs; whereas the dashed blue band presents the range of one standard error above and below the fitted line. Green-filled triangles schematically represent *Tyba* repeat arrays.

**Extended Data Fig. 6:**
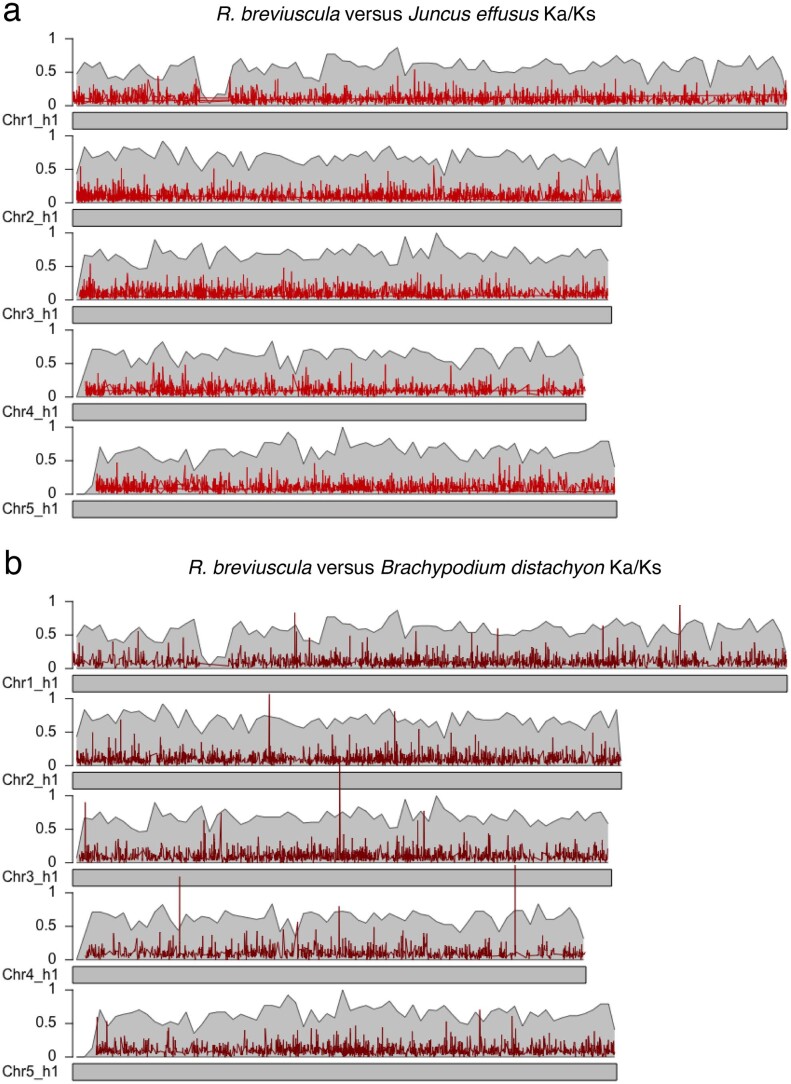
Ka/Ks ratio estimation across the chromosomes of *R. breviuscula*. **(a)** Ka/Ks ratio comparison between *R. breviuscula* and *Juncus effusus* genomes. **(b)** Ka/Ks ratio comparison between *R. breviuscula* and *Brachypodium distachyon* genomes. X-axis: gene start, Y-axis: Ka/Ks values; grey background: gene density. High Ka/Ks values indicate fast-evolving regions.

The distally biased recombination landscape is ubiquitous across many eukaryotic species, and is typically explained by suppression of CO formation at pericentromeric regions and an association with large-scale epigenetic regulation^20,21^. Hence, we set out to correlate the recombination landscape of *R. breviuscula* with several (epi)genetic features. Surprisingly, a chromosome-wide comparison revealed no apparent correlation of CO rates with the even distribution of hundreds of repeat-based centromeric units and other genomic (genes, transposable elements (TEs), SNP densities or GC content) and epigenomic (such as H3K4me3, H3K27me3, H3K9me2 or DNA methylation) features (Fig. 4a and Extended Data Fig. 5a). Further, genome-wide comparison of the *K*_a_/*K*_s_ ratio (a measurement of the relative rates of synonymous (*K*_s_) and non-synonymous (*K*_a_) substitutions at each gene) failed to identify fast-evolving genes correlated with higher recombination frequency regions (Extended Data Fig. 6). Quantification at the megabase scale of (epi)genomic features also revealed no strong correlation with CO distribution (Fig. 4b). These results indicate that, at the broad scale, meiotic recombination occurs independently of any (epi)genomic feature and chromosome-wide distribution of repeat-based holocentromeres.

### Lack of centromere effect and the fine-scale CO regulation

Next, we tested whether COs are epigenetically affected at a fine scale and whether individual centromeric units have an effect on CO designation in *R. breviuscula*. For that we used only the set of 378 COs resolved at high resolution (median 334 bp, mean ~2 kb) from our selfing offspring experiment.

**Extended Data Fig. 7:**
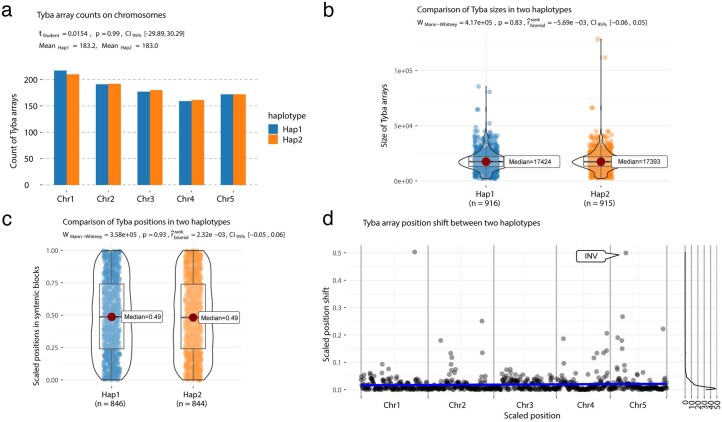
Structural variation among *Tyba* arrays between haplotype 1 and 2 in the phased genome of *R. breviuscula*. **(a)** Number of *Tyba* arrays on each chromosome of two haplotypes. Since *Tyba* array numbers in both haplotypes are normally distributed (Shapiro-Wilk normality test p-values: 0.7571 and 0.945) and have no significant difference in variance (F-test p-values: 0.767), two-sided t-test was performed to compare the means of *Tyba* array number of two haplotypes. **(b)** Sizes of *Tyba* of arrays in two haplotypes. Mean sizes of *Tyba* arrays in two haplotypes are 17,424 bp and 17,393 bp. N shows the number of *Tyba* arrays in each haplotype. The box plots in figure b-c are comprised of minima, Q1, median, Q3, and maxima following the definition of ggplot2 in R. Two-sided Mann-Whitney U tests were performed to compare the *Tyba* sizes (panel **b**) and scaled positions in syntenic blocks (panel **c**) between two haplotypes. **(c-d)** Scaled position distribution in two haplotypes (panel **c**) and position shift between haplotypes (panel **d**) after pairing *Tyba* arrays by the closest relative positions in the corresponding synteny blocks. N in panel **c** shows the number of paired syntenic *Tyba* arrays in two haplotypes. Most outliers in panel **d** are from inversion syntenic blocks as the callout shown on Chr5, long indels, and highly divergent regions based on SyRI output (see **Methods**).

The holocentromeres in *R. breviuscula* are repeat-based; that is, each centromeric unit is based on a specific array of the holocentromeric repeat Tyba associated with CENH3, with average sizes of ~20 kb and average spacings of ~400 kb, where each chromosome harbours hundreds of individual centromeric units (Extended Data Fig. 5b,c). Remarkably, we found the same epigenetic centromere identity in *R. breviuscula* (Extended Data Fig. 5d) as reported for *R. pubera*^32^. This organization makes it possible to identify centromeric units at the DNA level by annotating Tyba repeat arrays (Extended Data Fig. 5c). We then computed the observed versus expected by random distribution fine-scale CO position across all available chromatin marks and genetic features. We found that COs are formed more frequently at H3K4me3 peaks and genes than expected by random distribution (Fig. 4c and Supplementary Fig. 11). Within genic regions, COs were preferentially formed at the promoter regions (Fig. 4d). Remarkably, COs were mostly suppressed inside the core of centromeric units and heterochromatic regions (Fig. 4c–e, Extended Data Fig. 5e and Supplementary Fig. 11). Detailed comparison of homologous Tyba arrays between the two haplotypes revealed a lack of extensive structural variations (Extended Data Fig. 7). Thus, CO suppression within centromeric units is likely caused by epigenetic features, where high levels of DNA methylation are typically found (Extended Data Fig. 5d). Furthermore, after computing the distances between the CO break intervals and the corresponding nearest CENH3 domains/Tyba arrays, the COs did not show a tendency to be positioned away from or close to centromeric units (Fig. 4e,f and Extended Data Fig. 5e,f), indicating that proximity to a centromeric unit does not affect CO formation. Moreover, we found five cases of a CO being placed in a region containing reduced CENH3-positive chromatin and Tyba repeats (Fig. 4g), confirming the absence of a centromere effect in its proximity. Our results point to the exciting finding that local CO formation in *R. breviuscula* is abolished at repeat-based centromeric units but enriched at genic promoter regions, supporting the role of chromatin features at a fine scale in contrast to the absence of correlation at a broad scale.

### Telomere-led spatiotemporal dynamics of chromosome synapsis

**Fig. 5:**
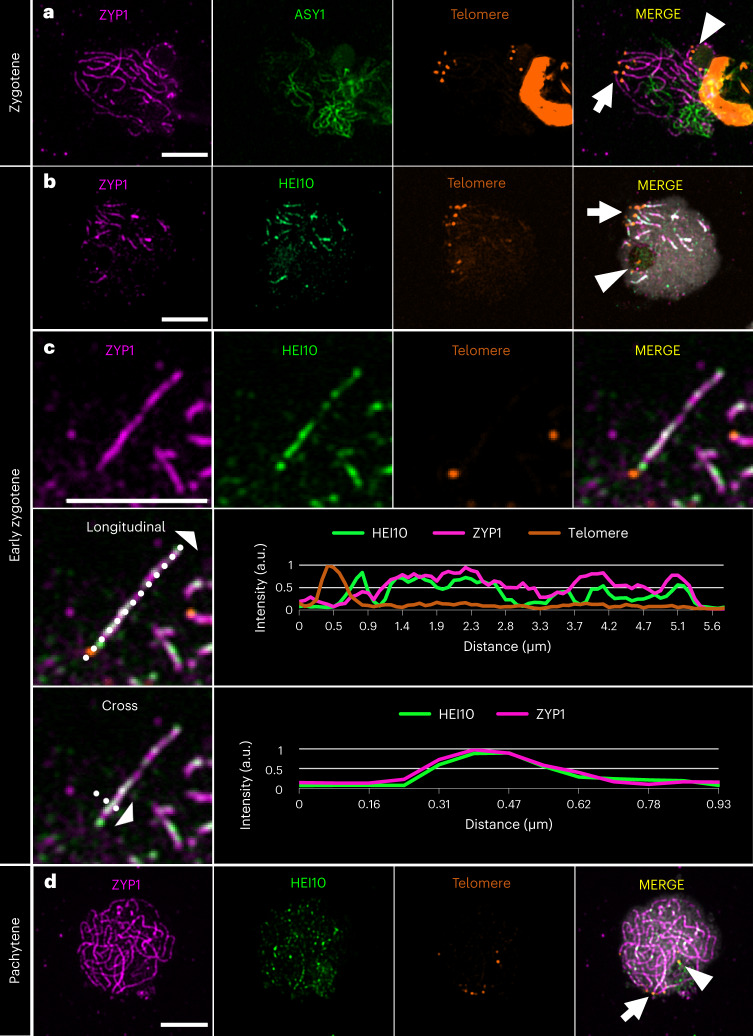
Telomere-led dynamics of synapsis formation and HEI10 loading. **a**, Telomeres cluster in a bouquet on one side of the cell, from where ZYP1 is elongating as the SC is being assembled. ASY1 represents unpaired chromosomes not yet reached by ZYP1. The behaviour of ASY1 + ZYP1 + telomeres was consistent in all immuno-FISH cells (*n* = 8) in three independent experiments. **b**, As ZYP1 lines elongate from the bouquet, HEI10 is quickly loaded onto synapsed chromosomes, whereas some telomeres localize to the nucleolus and lack the ZYP1 and HEI10 signals. **c**, Detail of early synapsis initiation and intensity profile of HEI10, ZYP1 and telomere: as soon as the SC (ZYP1) is assembled from telomeres, HEI10 is loaded and shows a high co-localization profile with ZYP1. a.u., arbitrary units. **d**, In late pachytene, ZYP1 occupies the whole chromosomal length, HEI10 signals become more dot-like and telomeres remain clustered in the bouquet, whereas few telomeres remain at the nucleolus. The behaviour of HEI10 + ZYP1 + telomeres was consistent in all immuno-FISH cells (*n* = 44) in three independent experiments. Telomeres at the bouquet (white arrow) and at the nucleolus (white arrowhead) are shown. Scale bar, 5 µm.

**Extended Data Fig. 8:**
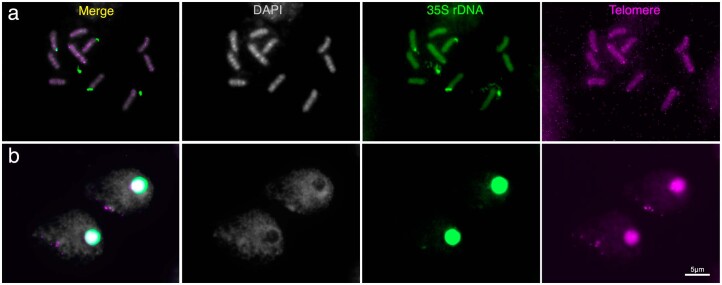
FISH with 35S rDNA and a telomeric probe in *R. breviuscula*. **(a)** Mitotic metaphase and **(b)** Prophase I. Telomeres of the *rDNA*-harbouring chromosomes 1 and 2 cluster in the nucleolus. Squares in **b** show telomeric sequences in chromosomes with 35 rDNA. FISH with 35S rDNA and telomeres showed consistent results (n = 25 cells) in two different experiments. Scale bar, 5 µm.

We hypothesized that pairing and synapsis contribute to the distally biased recombination landscape observed in *R. breviuscula*. To investigate this, we performed immunolocalization on meiocytes with antibodies against ZYP1, ASY1 and HEI10, and fluorescence in situ hybridization (FISH) for telomeres. Signals detected for ZYP1, ASY1 and telomere probes indicated a tendency for telomeric signals to cluster together in one location, forming a typical bouquet configuration^59–61^. We found that ZYP1 loading likely started preferentially from the bouquet (Fig. 5 and Supplementary Fig. 12). The linear signal of ASY1 was still present and represented unpaired chromosomes on the opposite cell side to the bouquet (Fig. 5a and Supplementary Fig. 13). Next, we asked whether HEI10 loading also shows telomere-led dynamics. Indeed, we could determine that the first synapsed regions (ZYP1-stained) were also first loaded with HEI10 in the proximity of chromosome ends (Fig. 5b,c). These early ZYP1 signals at sub-telomeric regions showed positive and non-random co-localization with early loaded HEI10 signals, but this correlation decreased as HEI10 signals became less linear and more dot-like throughout pachytene (Fig. 5c,d and Supplementary Fig. 14). We consistently observed a few telomeres that did not participate in the meiotic bouquet (*n* = 44). These signals represent the terminal ends of chr1 and chr2 that harbour the *35S rDNA* loci and show low recombination rates; instead, these chromosome ends localized in the nucleolus (Fig. 3a,b and Extended Data Fig. 8). Remarkably, the nucleolus-positioned telomeres showed delayed ZYP1 loading compared with the telomeres involved in the bouquet (Fig. 5b–d and Supplementary Fig. 13). Indeed, there was an average of four telomeric signals, consistent with two unsynapsed chromosome ends, whereas at the bouquet there was an average of eight telomeric signals, consistent with eight synapsed chromosome ends (*n* = 44). Thus, our results are compatible with telomere-led HEI10 loading on early synapsed chromosome ends, which seems to better explain the broad-scale recombination landscape in *R. breviuscula* rather than centromeric or (epi)genetic effects.

## Discussion

Deciphering the mechanisms controlling CO formation and distribution is key to understanding one of the main driving forces of genetic diversity in eukaryotes: meiotic recombination. Using *R. breviuscula*, a holocentric organism lacking both localized centromeres and compartmentalized chromosome organization, we show features that can potentially mask the factors underlying CO patterning and reveal important insights into CO control mechanisms. By showing that the CO distal bias observed in *R. breviuscula* is achieved even in the absence of compartmentalized chromosomal features, we propose that telomere-led (initiation at distal and sub-telomeric regions compared with more central regions) pairing and synapsis alone can impose bias in CO distribution. Indeed, the observed bouquet configuration has been shown to have a major role in synapsis and DNA DSB initiatiation^60–64^. Such telomere-led mechanisms have already been proposed to shift the concentration of COs towards the chromosome ends (Haenel et al.^22^ and references therein). The clustering of telomeres during early prophase I has been proposed to be responsible for telomere-led recombination in *Arabidopsis thaliana asy1* mutants and wheat^37,64^. However, we cannot exclude that other factors, such as the density of DSBs along chromosomes, might contribute to the observed distribution of COs^65^. Although our observations are compatible with synapsis elongating from the telomeres, we cannot infer the directionality of HEI10 loading. Moreover, a recent study has shown that the bouquet configuration is required for the distal bias of DSB formation and repair, possibly playing an important role in the observed reduced recombination rates at interstitial positions^66^. Future experiments in *Rhynchospora* will be important to identify conserved and adapted mechanisms of spatiotemporal dynamics of meiotic DSB formation and HEI10 loading.

**Fig. 6:**
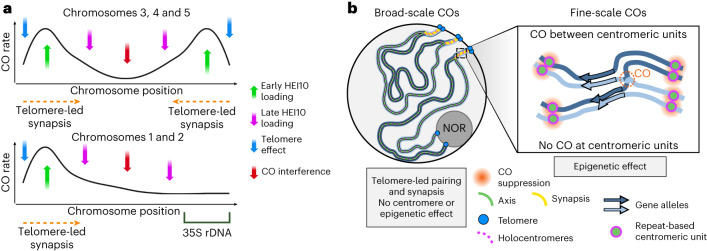
Model for CO regulation at a broad and fine scale in the holocentric plant *R. breviuscula*. **a**, Model for the role of telomere-led synapsis and HEI10 loading in shaping the broad-scale CO landscape in *R. breviuscula*. Whereas chr3, chr4 and chr5 have CO distal bias in both ends, chr1 and chr2 show bias at only one end, which is opposite to the localization of *35S rDNA* loci. **b**, Model for CO formation at a broad (left) and fine (right) scale. Telomere-led synapsis leads to the early loading of HEI10 at chromosomal ends that can potentially favour COs at distal regions, whereas 35S rDNA-harbouring chromosome ends do not show early synapsis and thus have less probability of making COs. At the local scale, COs are suppressed at core centromeric units, but not in their vicinity, where COs can be placed anywhere between two centromeric units. Remarkably, COs were preferentially placed at gene promoter regions.

Considering the conservation of bouquet formation and synapsis progression in *R. breviuscula*, we propose that telomere-led synapsis and HEI10 loading are the driving force that shapes the observed distal bias of COs, independent of any centromere effect. Recently, a ‘coarsening’ model has been proposed to describe CO interference, CO assurance and CO patterning^52,53^. This model is based on the ability of HEI10 to aggregate and diffuse along synapsed chromosomes. Interestingly, in this model, enhanced loading of HEI10 at the chromosome ends leads to increased COs. Because the amount of loaded HEI10 accounts for the increased coarsening over time, early loading at the chromosome distal regions would accelerate the maturation of recombination intermediates compared with the interstitial regions. Thus, the coarsening model, proposed as a conserved mechanism among eukaryotes, fits our observations. It also explains the observed reduction in COs at telomeric ends in our genetic analyses. Although the presence of unidentified HEI10 interactors contributing to a bias in its loading at specific regions cannot be discarded. Moreover, we found low recombination frequencies at the 35S rDNA-harbouring ends of chr1 and chr2. We observed that these telomeres do not participate in bouquet formation and are consequently subject to late synapsis and HEI10 loading. In *A. thaliana* rDNA localizes in the nucleolus, where it is shielded from synapsis and meiotic recombination^67,68^. Similarly, we propose that in *R. breviuscula*, chromosome ends harbouring 35S rDNA are involved late in synapsis, resulting in late HEI10 loading, delayed coarsening and finally lower recombination frequencies (Fig. 6).

In the new era of accurate long-read genomics, haplotype-phased genomes are routinely available. By applying high-throughput scRNA-seq to individual pollen nuclei, we provide a powerful pipeline to investigate CO frequencies in the gametes of any heterozygous individual with an available phased genome. Using haplotype-specific markers, we detected and mapped CO events from thousands of gametes in a species with repeat-based holocentromeres. Although the found distal bias of CO is similar to that observed in numerous eukaryotes, including the holocentric *C. elegans*^21,22,69^, the lack of correlation with the even distribution of (epi)genetic features (this study and Hofstatter et al.^32^) in *R. breviuscula* is remarkable. In *C. elegans* distinct chromosome domains (‘centre’ and ‘arms’) are characterized by differential gene density, repeats and histone modifications^21^, and the unequal CO distribution corresponds to its chromosome domains—recombination rates are lower at the centre than at the arms^70^. Furthermore, a recent study showed that the megabase-scale CO landscape in *A. thaliana* is mostly explained by association with (epi)genetic marks, with COs forming inside open chromatin^10^, and nucleotide polymorphisms only affecting COs locally^10,71^. By contrast, we could only find correlation of CO positioning with holocentromeres and (epi)genetic features at a fine scale, where COs preferentially formed within gene promoters rather than gene bodies, TEs and centromeres (Fig. 6b). This result appears to hold true for several eukaryotes and may be related to open chromatin states^9,10,72^, suggesting that fine-scale CO regulation is associated with similar (epi)genetic factors independent of the chromosome organization. By contrast, the absence of a centromere effect in *R. breviuscula*, which seems to suppress CO formation only inside centromeric units but not in their vicinity (Fig. 6b), is probably due to the closed chromatin state of centromeric chromatin in *R. breviuscula*, as marked by high DNA methylation levels. Our findings suggest that the pericentromeric inhibition of COs observed in many monocentric eukaryotes^12,22^ is likely a secondary effect of heterochromatin accumulation along large monocentric pericentromeres, and not a direct effect of centromeric chromatin. Understanding the molecular mechanisms of CO control in holocentric organisms will potentially unveil new strategies to address meiotic recombination within centromere proximal regions of monocentric chromosomes that rarely recombine.

## Methods

### Plant material

The same specimen of *Rhynchospora breviuscula* previously sequenced^32^ was propagated clonally and cultivated in a greenhouse at the Max Planck Institute for Plant Breeding Research in Cologne, Germany. Flower buds were used for screening meiotic cells and pollen was collected for single-gamete sequencing.

### Isolation of pollen nuclei, 10x Genomics scRNA-seq library preparation and sequencing

Protocols were adapted from Campoy et al.^58^. Briefly, to release pollen grains, anthers from fully developed flowers of *R. breviuscula* and *R. tenuis* (for multiplex purposes) were harvested and submerged in woody pollen buffer^73^. The nuclei were extracted using a modified bursting method. The solution containing the pollen grains was pre-filtered with a 100 µm strainer, and the pollen was crushed on a 30 µm strainer (Celltrics). The isolated nuclei were gathered in woody pollen buffer and stained with 4,6-diamidino-2-phenylindole (DAPI; 1 µg ml^−1^) before being sorted using a BD FACSAria Fusion sorter with a 70 µm nozzle and 483 kPa sheath pressure. A total of 10,000 nuclei were sorted into 23 µl of sheath fluid solution and loaded into a 10x Chromium controller, according to the manufacturer’s instructions. A library was created according to the chromium single-cell 3′ protocol. A CG000183 https://www.ncbi.nlm.nih.gov/nuccore/CG000183 Rev A kit from 10x Genomics was used for library preparation. The library was sequenced (100 Gb) on an Illumina NovaSeq instrument in 150 bp paired-end mode.

### Whole-genome sequencing of F_1_ recombinant offspring

To obtain a recombinant population of *R. breviuscula* plants, young inflorescences of the heterozygous reference *R. breviuscula* were bagged to force self-pollination. Because of its high self-incompatibility, only 63 seeds were obtained and germinated in soil to obtain F_1_ plants. All 63 F_1_ plants were sequenced to 3× coverage (~2 Gb) using an Illumina NextSeq2000 instrument in 150 bp paired-end mode.

### Anther fixation and immunocytochemistry

Immunostaining was performed as described by Marques et al.^31^ with some modifications. Anthers of *R. breviuscula* were harvested and fixed in ice-cold 4% (w/v) paraformaldehyde in phosphate-buffered saline (PBS; pH 7.5, 1.3 M NaCl, 70 mM Na_2_HPO_4_, 30 mM NaH_2_PO_4_) for 90 min. The anthers were separated according to size and dissected to release the meiocytes onto glass slides. The meiocytes were squashed with a coverslip that was later removed using liquid nitrogen. Meiocytes were then blocked with 1 h incubation in 3% (w/v) bovine serum albumin in PBS + 0.1% (v/v) Triton X-100 at 37 °C. The antibodies used were anti-AtASY1 raised in rabbits (inventory code PAK006)^36^, anti-AtMLH1 raised in rabbits (PAK017)^74^ and anti-*Rhynchospora*CENH3 raised in rabbits^30^. The anti-ZYP1 was raised in chickens against the peptide EGSLNPYADDPYAFD of the C-terminal end of AtZYP1a/b (gene ID: At1g22260/At1g22275) and affinity-purified (Eurogentec) (PAK048). Remarkably, this peptide region showed 100% similarity with the *Rhynchospora* ZYP1 C terminus (gene ID: RBREV_HAP1.r01.Chr2_h1G00222020.1). The anti-REC8 was a combination of two antibodies raised in rabbits against the *Rhynchospora*-specific REC8-peptides C-EEPYGEIQISKGPNM and C-YNPDDSVERMRDDPG (gene ID: RBREV_HAP1.r01.Chr4_h1G00395720.1) and affinity-purified (Eurogentec). The anti-HEI10 was a combination of two antibodies raised in rabbits against the *Rhynchospora*-specific HEI10-peptides C-NRPNQSRARTNMFQL and C-PVRQRNNKSMVSGGP (gene ID: RBREV_HAP1.r01.Chr4_h1G00387160.1) and affinity-purified (Eurogentec). Each primary antibody was diluted 1:200 in blocking solution. The slide-mounted samples were incubated with the primary antibodies overnight at 4 °C, after which they were washed three times for 10 min with PBS + 0.1% (v/v) Triton X-100. The slides were incubated with the secondary antibodies for 2 h at room temperature. The secondary antibodies were conjugated with Abberior STAR ORANGE (dilution 1:250; goat anti-rabbit immunoglobulin (Ig)G, catalogue no. STORANGE-1002-500UG, or goat anti-chicken IgY, catalogue no. STORANGE-1005-500UG) or Abberior STAR RED (dilution 1:250, goat anti-rabbit IgG, catalogue no. STRED-1002-500UG or goat anti-chicken IgY, catalogue no. STRED-1005-500UG) before being washed again three times for 10 min with PBS + 0.1% (v/v) Triton X-100 and allowed to dry. The samples were prepared with 10 µl of mounting solution (Vectashield + 0.2 µg of DAPI), covered with a coverslip and sealed with nail polish for storage. Images were taken with a Zeiss Axio Imager Z2 with Apotome system for optical sectioning or with a Leica Microsystems Thunder Imager dMi8 with computational clearing. The images were deconvolved and processed with ZEN 3.2 or LAS X software. Co-localization analysis of ZYP1 and HEI10 signals was performed using the co-localization function of ZEN 3.2 software (Zeiss) and auto-thresholding was done using the Costes function^75,76^.

### Sequential immunostaining and fluorescence in situ hybridization

Sequential immunostaining and fluorescence in situ hybridization (immuno-FISH) was performed following Baez et al.^77^. The best slides obtained from immunostaining, as described above, were selected for FISH using a telomeric probe. Slides were washed with 1× PBS for 15 min, postfixed in 4% (w/v) paraformaldehyde in PBS for 10 min, dried with 70% (v/v) and 100% ethanol for 5 min each and probed with direct-labelled telomeric sequence (Cy3-[TTTAGGG]_5_; MilliporeSigma). The hybridization mixture contained formamide (50% w/v), dextran sulfate (10%, w/v), 2× saline-sodium citrate (SSC) buffer and 50 ng μl^−1^ of telomeric probe. The slides were denatured at 75 °C for 5 min. Stringency washes were performed following Braz et al.^78^ to give a final stringency of approximately 72%. The slides were counterstained with 10 µl of mounting solution (Vectashield + 0.2 µg of DAPI), and images were captured as described above.

Mitotic and meiotic chromosome spreads were performed as described by Ruban et al.^79^, with some modifications. Briefly, tissue samples were fixed in a 3:1 ethanol/acetic acid (v/v) solution for 2 h with gentle shaking. The samples were washed with water twice for 5 min and treated with an enzyme mixture (0.7% w/v cellulase R10, 0.7% w/v cellulase R10, 1.0% w/v pectolyase and 1.0% w/v cytohelicase in citric buffer) for 30 min at 37 °C. The samples were dissected on slides under a binocular microscope in acetic acid (60%). The pTa71 probe (18S–5.8S–26S ribosomal RNA) from *Triticum aestivum*^80^ was labelled with Alexa-448 by nick translation for 35S rDNA localization. FISH with telomeric and 35S rDNA probes was performed as described above.

### Haplotype phasing and scaffolding

A phased chromosome-level genome of *R. breviuscula* was assembled using PacBio HiFi and Hi-C data available from Hofstatter et al.^32^ under the NCBI BioProject no. PRJNA784789. First, a phased primary assembly was obtained by running hifiasm^81^ using as inputs the 30 Gb of PacBio HiFi reads (~35× coverage per haplotype) in combination with Dovetail Omni-C reads, using the following command: hifiasm -o Rbrevi.phased.asm.hic ––h1 hic.R1.fastq.gz ––h2 hic.R2.fastq.gz hifi.reads.fastq.gz. The phased assemblies of each individual haplotype were further scaffolded to chromosome scale using Salsa2 (ref. ^82^), followed by successive rounds of manual curation and rescaffolding. The genome sizes of haplotypes 1 and 2 were 418,624,405 and 390,890,712 bp, respectively. Both haplotypes comprise five chromosomes with a length of ~370 Mb in total, as well as other unplaced sequences (Supplementary Table 1).

### Hi-C map generation and haplotype comparison

The Hi-C heatmap (Extended Data Fig. 3c) was generated with juicer (v.1.6) by aligning Omni-C reads that were used for genome assembly to the phased *R. breviuscula* genome. The Hi-C triangles for each chromosome in Fig. 4a and Extended Data Fig. 5a were plotted using fancplot (v.0.9.1) with 500,000bp resolution and Knight-Ruiz (KR) normalization. Synteny blocks and structural rearrangements between two haplotypes (Extended Data Fig. 3d) were computed by SyRi (v.1.5.3) and plotsr^83,84^ after aligning two haplotypes by minimap2 (v.2.20).

### Definition of allelic SNPs as genotyping markers on the phased reference genome

To define genotyping markers for *R. breviuscula*, all available (NCBI BioProject no. PRJNA784789) raw Illumina HiSeq 3000 150 bp paired-end reads (25,899,503,075 bases, ~54× coverage) were first mapped to the five pseudochromosome scaffolds in haplotype 1 of the phased reference genome using bowtie2 (v.2.4.4)^85^. The alignment file was further sorted with SAMtools (v.1.9)^86^. The alignments of short reads to the reference genome were used for SNP calling by ‘bcftools mpileup’ and ‘bcftools call’ (v.1.9)^86^ (with the –keep-alts, ––variants-only and ––multiallelic-caller flags enabled). A total of 1,404,927 SNPs excluding insertions/deletions were derived. To distinguish the two haplotypes using these SNPs, only allelic SNPs were selected as markers for genotyping; therefore, variant information was collected, including mapping quality, alternative base coverage and allele frequency resulting from SHOREmap conversion (v.3.6)^87^, which converts SNP files (.vcf) into a read-friendly, tab-delimited text file. A final set of 820,601 alleles fulfilling certain thresholds (mapping quality >50; 5 ≤ alternative base coverage ≤ 30, 0.4 ≤ allele frequency ≤ 0.6) was selected as markers (Fig. 2b and Supplementary Fig. 6).

### Preprocessing scRNA-seq data from pollen nuclei

Raw scRNA-seq data usually include barcode errors and contaminants such as doublets and ambient RNA. In the current study, cell barcodes (CBs) were first corrected in these data using ‘bcctools correct’ (v.0.0.1) based on the 10x v3 library complete barcode list with options ‘––alts 16 ––spacer 12’ because of the 16 bp CB and 12 bp unique molecular identifier (UMI). After correction, 952,535 viable CBs were detected. This step also truncated the CBs and UMIs from every pair of scRNA-seq reads. After counting the occurrence of CBs, the number of read pairs under each CB was determined. To ensure a sufficient number of reads for SNP calling, only CBs appearing more than 5,000 times were used for the subsequent analyses. Finally, each CB was seen as one viable cell, and reads corresponding to the CB were assigned to this cell (demultiplexing). A total of 8,001 viable cells were ultimately identified, with 365,771,748 (77.25% of all raw scRNA-seq) read pairs included. We also input the scRNA-seq data to the 10x standard analysis pipeline cellranger (v.7.1.0) to check the statistics. The clustering analysis and the gene number in each cell (Supplementary Fig. 5) were based on cellranger count results.

### Alignments of single-pollen RNA sequences to genome and deduplication

To identify genotyping markers in the *R. breviuscula* gametes, scRNA reads of the pollen nuclei were first mapped to the haplotype 1 chromosomes (Fig. 2b) using hisat2 (v.2.1.0)^88^. Specifically, each cell-specific pair of reads was merged as one single-end FASTQ file, and hisat2 was run under single-end mode (-U) because the SNP calling approach does not detect SNPs on reads whose mated reads are not mapped. Before further analyses of the alignment results, UMIs were previously extracted from the read alongside the CBs; hence, a fast UMI deduplication tool, UMIcollapse^89^, was employed to remove the PCR duplicates by collapsing reads with the same UMIs.

The sequencing library was prepared for mixed pollen nuclei of *R. breviuscula* and *R. tenuis* to enable multiple-potential analyses. The addition of gametes from *R. tenuis* was done for multiplexing purposes, and they are analysed in another study. To discriminate the single-cell data between the two species, we used a straightforward approach without gene expression profiling. For each cell: (1) the DNA sequences were mapped to both the *R. breviuscula* and *R. tenuis* chromosomal genomes; and (2) the alignment rates between the two species were compared to decide the cell identity (Supplementary Fig. 7). The alignment rates to *R. breviuscula* and *R. tenuis* were both bimodal distributions (Supplementary Fig. 7a,b); therefore, these cells can be grouped based solely on their mapping rates. It was estimated that 4,733 cells were from *R. breviuscula* and 2,709 cells were from *R. tenuis* (Supplementary Fig. 7c) based on the alignment fractions. The remaining 559 cells presented very similar alignment rates, which were potential doublets. Among the 4,733 *R. breviuscula* cells, those whose alignment rates were lower than 25% were discarded, leaving 4,392 cells from *R. breviuscula* available for the next stage of the analysis.

### SNP calling and selection of markers in gametes

SNP calling in all gametes adopted the same methods as the reference genome SNP calling; for example, via ‘bcftools mpileup’ and ‘bcftools call’ (v.1.9), with the difference that the ‘––variants-only’ flag was not applied. After acquiring SNPs for every gamete, the SNP positions, allele counts of the reference and alternative bases were extracted through the ‘bcftools query’. Comparing SNPs in every gamete with markers defined on the reference resulted in reliable genotyping markers in this gamete.

A total of 2,338 cells with fewer than 400 markers were first discarded to ensure accurate genotyping by sufficient markers. To remove doublets, the frequency of marker genotype switches across the remaining 2,054 cells was estimated. Cells with frequent switches—that is, a switching rate (genotype switching times/number of markers) greater than 0.07—were taken as doublets (Supplementary Fig. 7e). Ultimately, 402 doublets were identified, with the remaining 1,652 cells proving suitable for subsequent CO calling.

### CO identification

Chromosome genotyping was performed by adapting the haplotype phasing method proposed by Campoy et al. The original approach was designed based on a scDNA-seq library that is commonly able to examine more SNPs than scRNA-seq data. Therefore, the smoothing function and parameters were adjusted accordingly to genotype genomic blocks with relatively sparse markers. Specifically, the markers were first smoothed using the allele frequencies of neighbouring markers (two ahead and two behind), and then smoothed by the genotypes of surrounding markers. After smoothing, the genomic segments harbouring the markers in the same genotype are merged to genotype blocks, and those containing at least five markers within 1 Mb length were qualified to be assigned a final genotype. The genomic regions that saw the conversion of the genotypes at the flanks were taken as CO break positions (Fig. 2c,d). Finally, the CO numbers in each cell were counted, and those with double COs were manually assessed and corrected.

### Recombination landscape and CO interference from single-gamete sequencing

To gain an overview of the CO rates across the chromosomes of *R. breviuscula*, the CO positions in all viable cells (1,641 cells remaining after manual correction) were summarized, and the recombination landscape for each chromosome was plotted (Fig. 3a). The recombination rate (cM Mb^−1^) was computed using a 1 Mb sliding window and 100 kb step size.

CO interference was analysed with MADpattern (v.1.1)^90^, using 1,641 confident singleton pollen nuclei. Chromosome 1 was divided into 18 intervals and chr2–chr5 were divided into 15 intervals to compute the mean CoC of every pair of intervals.

### F_1_ offspring mapping and CO analysis

Sixty-three F_1_ offspring were reproduced from selfed *R. breviuscula*. Each F_1_ plant was sequenced with ~3× Illumina whole-genome sequencing data. To genotype F_1_ offspring, whole-genome sequencing Illumina sequences of each plant were first mapped to the rhyBreHap1 reference genome using bowtie2 (v.2.4.4) paired-end mode, and then SNPs were called by ‘bcftools mpileup’ and ‘bcftools call’ (v1.9) (with ––keep-alts, ––variants-only and ––multiallelic-caller flags enabled). Next, SNPs of each F_1_ sample were input to TIGER^91^ for genotyping and to generate potential CO positions. In addition, RTIGER^92^ was used to identify the genotypes of chromosomal segments by utilizing the corrected markers that resulted from TIGER. Only COs that agreed using both tools were kept. The recombination landscape from F_1_ COs (Supplementary Fig. 4) was plotted using the same strategy and sliding window, as illustrated for pollen nuclei.

### Genetic linkage maps

To plot the genetic linkage maps (Fig. 3b and Extended Data Fig. 4a), 743 markers were extracted from the 820,601 reference markers by selecting the median marker within each 500 kb sliding window (step size was also 500 kb) from the first present marker to the last. The linkage map was then plotted using R package LinkageMapView. Marey maps (Fig. 3c and Extended Data Fig. 4b) were plotted using genetic length, computed based on pollen nuclei with more than 2,000 markers (*n* = 81) and F_1_ offspring (*n* = 63), against physical length for each chromosome, respectively.

### Chromatin immunoprecipitation

CENH3 chromatin immunoprecipitation sequencing (ChIP–seq) data were obtained from Hofstatter et al.^32^. Further chromatin immunoprecipitation (ChIP) experiments were performed for H3K4me3 (rabbit polyclonal to histone H3 tri-methyl K4; Abcam, catalogue no. ab8580, dilution 1:300), H3K9me2 (mouse monoclonal to histone H3 di-methyl K9; Abcam, catalogue no. ab1220, clone no. mAbcam 1220, dilution 1:200), H3K27me3 (mouse monoclonal to histone H3 tri-methyl K27; Abcam, catalogue no. ab6002, clone no. mAbcam 6002, dilution 1:200) and the IgG control (recombinant rabbit IgG, monoclonal; Abcam, catalogue no. ab172730, clone no. EPR25A, dilution 1:300) using the protocol described by Hofstatter et al.^32^.

### ChIP–seq and analysis

ChIP DNA was quality-controlled using the next-generation sequencing assay on a FEMTO pulse (Agilent Technologies). An Illumina-compatible library was prepared with the Ovation Ultralow V2 DNA-Seq library preparation kit (Tecan Genomics) and sequenced as single-end 150 bp reads on a NextSeq2000 (Illumina) instrument. An average of 20 million reads were obtained for each library.

Raw sequencing reads were trimmed using Cutadapt^93^ to remove low-quality nucleotides (with a quality score <30) and the adaptors. Trimmed ChIPed 150 bp single-end reads were mapped to their respective reference genome using bowtie2 (ref. ^85^) with default parameters. All read duplicates were removed and only the single best-matching read was kept on the final alignment Binary Alignment Map (BAM) file. The BAM files were converted to BIGWIG coverage tracks using the bamCompare tool from deeptools^94^. Coverage was calculated as the number of reads per 50 bp bin and normalized as reads per kilobase per million mapped reads (RPKM). Magnified chromosome regions showing multiple tracks presented in Fig. 4g were plotted with pyGenomeTracks^95^.

### Tyba array and CENH3 domain annotation

Tyba repeats were annotated using a BLAST search with a consensus Tyba sequence, allowing a minimum of 70% similarity. Further annotation of the Tyba arrays was performed by removing spurious low-quality Tyba monomer annotations shorter than 500 bp. bedtools^96^ was used to merge all adjacent Tyba monomers situated at a maximum distance of 25 kb into individual annotations to eliminate the gaps that arise because of fragmented Tyba arrays, and those smaller than 2 kb were discarded.

CENH3 peaks were called with MACS3 (ref. ^97^) using the broad peak calling mode:macs3 callpeak -t ChIP.bam -c Control.bam ––broad -g 380000000 ––broad-cutoff 0.1

The identified peaks were further merged using a stepwise progressive merging approach. CENH3 domains were generated by: (1) merging CENH3 peaks with a spacing distance less than 25 kb using bedtools to eliminate the gaps that arise because of fragmented Tyba arrays or the insertion of TEs; and (2) removing CENH3 domains less than 1 kb in size.

### Transposable element annotation

TE protein domains and complete long terminal repeat retrotransposons were annotated in the reference haplotype genome using the REXdb database (Viridiplantae_version_3.0)^98^ and the DANTE tool available from the RepeatExplorer2 Galaxy portal^99^.

### Enzymatic methyl sequencing and analysis

To investigate the methylome space in *R. breviuscula*, the relatively non-destructive NEBNext Enzymatic Methyl-seq Kit was employed to prepare an Illumina-compatible library, followed by paired-end sequencing (2 × 150 bp) on a NextSeq2000 (Illumina) instrument. For each library, 10 Gb of reads was generated.

Enzymatic methyl sequencing data were analysed using the Bismarck pipeline^100^ following the standard pipeline described at https://rawgit.com/FelixKrueger/Bismark/master/Docs/Bismark_User_Guide.html. Individual methylation context files for CpG, CHG and CHH were converted into BIGWIG format and used as input tracks for the overall genome-wide DNA methylation visualization with pyGenomeTracks and R plots.

### Quantitative correlation of COs and (epi)genetic features

The correlation matrix (Fig. 4b) was calculated for all pairwise features by Pearson correlation coefficient using sliding window: specifically, mean CO rates, mean GC contents, CENH3 peak density, Tyba array density, SNP density, TE density, H3K4me3 RPKM, H3K9me2 RPKM, H3K27me3 RPKM, mean CpG, mean CHG and mean CHH.

To inspect a possible centromere effect on CO positioning, the relative distance from the CO site was calculated to the closest left and right centromeric unit (the CENH3 domain or Tyba array) across the 378 COs in the F_1_ offspring and normalized all distances to 0–1 such that all neighbouring centromeric units were displayed at the same scale (Fig. 4f, Extended Data Fig. 5f and Supplementary Fig. 11e,f). CO and marker positions over the transcript bodies, CENH3 domain or Tyba array were normalized by their distance to start sites and end sites and then counted by binning (Fig. 4d,e, Extended Data Fig. 5e and Supplementary Fig. 11).

To see the association of CO designations with a variety of (epi)genetic features at a local scale, we first counted the number of COs that overlap with CENH3, Tyba arrays, genes, TEs, long terminal repeats, H3K4me3 peaks, H3K9me2 peaks and H3K27me3 peaks using ‘bedtools intersect’ (v.2.29.0). Next, we assigned 378 pseudo-COs genome-wide at random. The number of COs on each chromosome was the same as that detected by F_1_ individuals (for example, 72 COs on chr1, 69 on chr2, 76 on chr3, 84 on chr4 and 77 on chr5), whereas the CO break gap length was picked up from the 378 real F_1_ CO gaps randomly. For each simulation round, the pseudo-COs were overlapped with (epi)genetic features, again using ‘bedtools intersect’. Five thousand of these simulations were done, and the results were then plotted as the distribution of overlapped CO numbers for each feature (Supplementary Fig. 11). Finally, to evaluate the deviation of real overlapped COs with each feature from the expected overlapped CO number under the hypothesis of randomly distributed COs, *Z*-scores were calculated using the mean values and standard deviations of the simulated number of overlapped CO distribution (Fig. 4c).

### Gene annotation

Structural gene annotation was done by combining de novo gene calling and homology-based approaches with *Rhynchospora* RNA-seq, IsoSeq and protein datasets already available^32^.

Using evidence derived from expression data, RNA-seq data were first mapped using STAR^101^ (v.2.7.8a) and subsequently assembled into transcripts by StringTie^102^ (v.2.1.5, parameters -m 150-t -f 0.3). Triticeae protein sequences from available public datasets (UniProt, https://www.uniprot.org, 5 October 2016) were aligned against the genome sequence using GenomeThreader^103^ (v.1.7.1; arguments -startcodon -finalstopcodon -species rice -gcmincoverage 70 -prseedlength 7 -prhdist 4). IsoSeq datasets were aligned to the genome assembly using GMAP^104^ (v.2018-07-04). All assembled transcripts from RNA-seq, IsoSeq and aligned protein sequences were combined using Cuffcompare^105^ (v.2.2.1) and subsequently merged with StringTie (v.2.1.5, parameters ––merge -m150) into a pool of candidate transcripts. TransDecoder (v.5.5.0; http://transdecoder.github.io) was used to identify potential open reading frames and to predict protein sequences within the candidate transcript set.

Ab initio annotation was initially done using Augustus^106^ (v.3.3.3). GeneMark^107^ (v.4.35) was additionally employed to further improve structural gene annotation. To avoid potential over-prediction, we generated guiding hints using the above-described RNA-seq, protein and IsoSeq datasets as described by Nachtweide and Stanke^106^ A specific Augustus model for *Rhynchospora* was built by generating a set of gene models with full support from RNA-seq and IsoSeq. Augustus was trained and optimized using the steps detailed by Nachtweide and Stanke^106^.

All structural gene annotations were joined using EVidenceModeller^108^ (v.1.1.1), and weights were adjusted according to the input source: ab initio (Augustus: 5, GeneMark: 2), homology-based (10). In addition, two rounds of PASA^109^ (v.2.4.1) were run to identify untranslated regions and isoforms using the above-described IsoSeq datasets.

We used DIAMOND^110^ (v.2.0.5) to compare potential protein sequences with a trusted set of reference proteins (UniProt Magnoliophyta, reviewed/Swiss-Prot, downloaded on 3 August 2016; https://www.uniprot.org). This differentiated candidates into complete and valid genes, non-coding transcripts, pseudogenes and TEs. In addition, we used PTREP (release 19; https://trep-db.uzh.ch). Furthermore, functional annotation of all predicted protein sequences was done using the AHRD pipeline (https://github.com/groupschoof/AHRD).

### *K*_a_/*K*_s_ ratio calculation

We identified homologues between *Brachypodium distachyon* (v.3.0) (downloaded from ensemble plants plants.ensembl.org) and *Juncus effesus*^32^ using the ortholog module from JCVI python library^111^. Subsequently, pairwise alignments were generated with ParaAT^112^ (v.2) and the *K*_a_/*K*_s_ ratio was calculated with KaKs_Calculator^113^ (v.3) using the YN method^114^. Plots were generated using karyoploteR^115^.

### Structural comparison of Tyba arrays between two haplotypes

The Shapiro test was applied to Tyba array numbers, which suggested a normal distribution of array number in both haplotypes. A two-sided *F*-test was then performed and proved the equality of their variances. Thus, we applied a two-sided *t*-test, which suggested no significant difference of means of Tyba array number in two haplotypes (*P* = 0.9881). The sizes of Tyba arrays on both haplotypes also showed no difference in median based on Mann–Whitney *U*-test (*P* = 0.83). Next, we compared the relative positions of Tyba arrays within the colinearly syntenic blocks between haplotypes; that is, the position of the midpoint of each Tyba array was scaled to the syntenic block in which it resides. A two-sample Kolmogorov–Smirnov test was performed on this relative Tyba array position in two haplotypes, which implied that both followed the same distribution (*D* = 0.021694, *P* = 0.9886). We ended up with 823 pairs when further linking the Tyba arrays between two haplotypes that reside at the closest relative syntenic positions. Linear regression demonstrated that the difference in the relative positions of those Tyba array pairs almost fit to a horizontal line of *y* = 0 indicating that the relative positions of paired Tyba arrays in syntenic counterparts are mostly the same despite a few outliers (Extended Data Fig. 7).

### Reporting summary

Further information on research design is available in the Nature Portfolio Reporting Summary linked to this article.

### Supplementary information

Supplementary Information Supplementary Tables 1 and 2, and Figs. 1–14.

Reporting Summary

## Data Availability

All sequencing data used in this study have been deposited at NCBI under the BioProject ID PRJNA1059790 and are publicly available as of the date of publication. The reference genomes, sequencing data, annotations and all tracks presented in this work are made available for download at DRYAD: https://datadryad.org/stash/share/EvB3PRNVph5IiTkOM3jTZddgmS45cJhQYq2v3LI5InE. The REXdb database Viridiplantae v.3.0 [http://repeatexplorer.org/?page_id=918] is publicly available. All other data needed to evaluate the conclusions in the paper are provided in the paper and/or the supplemental information.

## Code availability

The original code for the construction of recombination maps from single-cell RNA sequencing is available at https://github.com/Raina-M/detectCO_by_scRNAseq. Any additional information required to re-analyse the data reported in this paper is available from the corresponding author upon request.
