# Supplementary material for: Meiotic recombination dynamics in plants with repeat-based holocentromeres shed light on the primary drivers of crossover patterning
Source: Nat Plants. 2024 Feb 9;10(3):423–38. doi: 10.1038/s41477-024-01625-y (PMC10954556; doi:10.1038/s41477-024-01625-y)
Supplement: Supplementary file 1 — Supplementary Tables 1 and 2, and Figs. 1–14. [file 41477_2024_1625_MOESM1_ESM.pdf]

nature plants

Article

https://doi.org/10.1038/s41477-024-01625-y

Meiotic recombination dynamics in plants with repeat-based holocentromeres shed light on the primary drivers of crossover patterning

In the format provided by the authors and unedited

Supplementary Table 1. Summary of genome size, contigs, and scaffolds of the phased genome assemblies.

	Haplotype 1	Haplotype 2
**Genome assembly size (bp)**	418,627,160	390,890,803
**# Contigs**	1637	548
**Contig assembly size (bp)**	421,256,472	391,742,506
**Largest contig (bp)**	35,313,519	43,961,622
**Contig N50 (bp)**	11,938,939	13,764,201
**Contig N90 (bp)**	42,248	2,739,863
**# Scaffolds**	1,501	457
**Pseudo-chromosome size (bp)**	368,174,147	370,478,156
**Scaffold N50 (bp)**	69,585,868	72,168,595
**Scaffold N90 (bp)**	45,843	66,381,717
**Largest scaffold / chr 1 (bp)**	91,632,052	89,220,796
**Chromosome 2 (bp)**	70,953,004	72,168,595
**Chromosome 3 (bp)**	69,585,868	69,956,709
**Chromosome 4 (bp)**	66,447,897	66,381,717
**Chromosome 5 (bp)**	69,555,326	72,750,339
**Base accuracy (QV)**	30.85	32.32
**Completeness (%)**	85	85
**GC (%)**	35.91	35.60

Supplementary Table 2. Synteny and structural variations between two haplotypes of *R. breviuscula*.

#Structural annotations			
#Variation_type	Count	Length hap1	Length hap2
**Syntenic regions**	229	329,130,991	329,924,075
**Inversions**	39	2,135,010	1,947,035
**Translocations**	346	3,620,755	3,569,626
**Duplications (reference)**	137	1,472,557	–
**Duplications (query)**	249	–	1168366
**Not aligned (reference)**	650	32,783,105	–
**Not aligned (query)**	808	–	33606738
#Sequence annotations			
#Variation_type	Count	Length hap1	Length hap2
**SNPs**	615,883	615883	615,883
**Insertions**	59,142	–	2,687,428
**Deletions**	59,276	3,101,459	–
**Copy gains**	87	–	126,950
**Copy losses**	60	394,961	–
**Highly diverged**	5,660	172,894,800	174,131,686
**Tandem repeats**	3	482	825

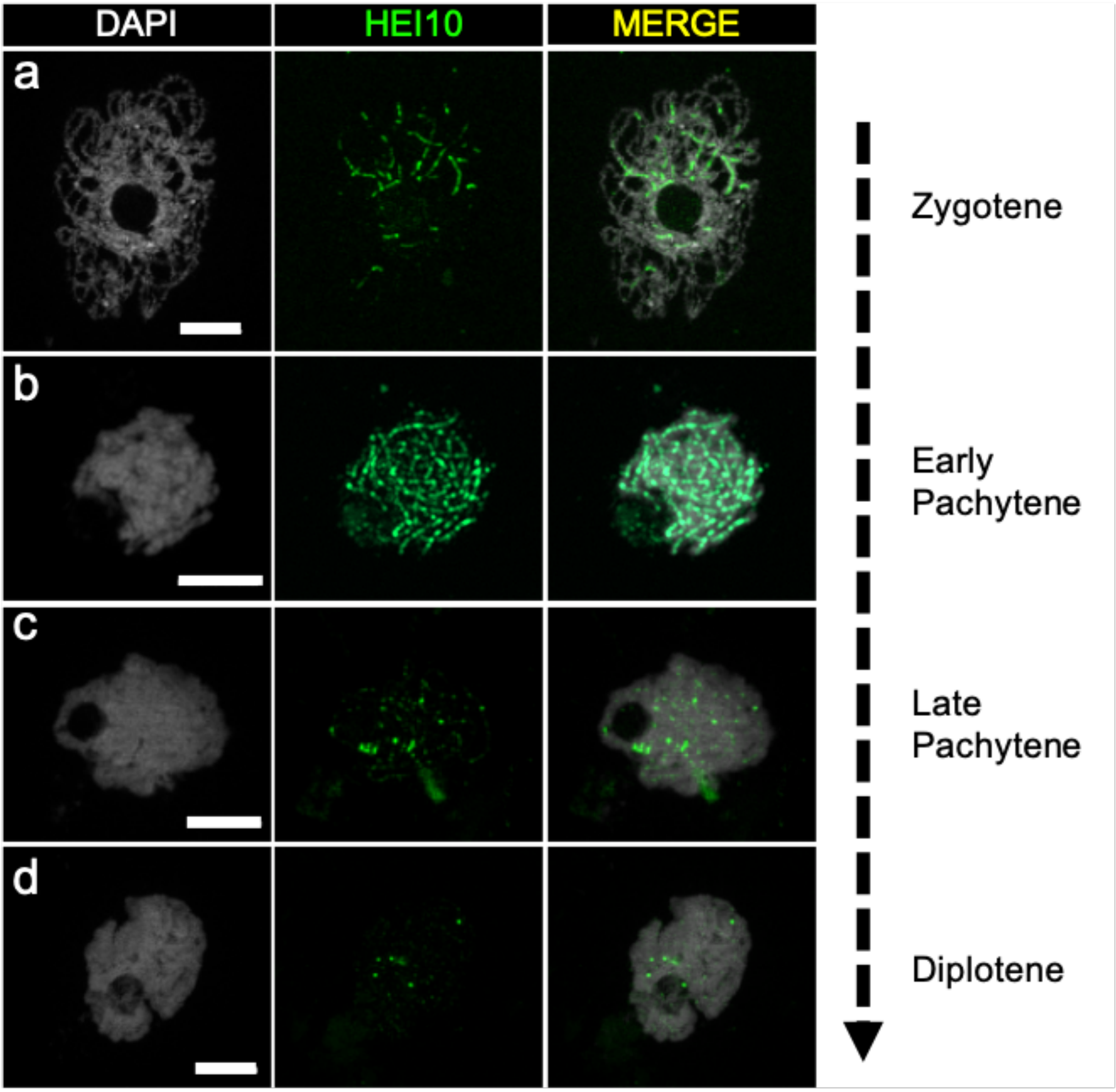

Supplementary Fig. 1: Dynamic pattern of HEI10 through Prophase I. (a) At early zygotene, HEI10 is loaded on synapsed chromosomes as a linear signal made of closely spaced dots. **(b)** At early pachytene, when synapsis is complete, the linear dotty signal can be observed covering the entire length of synapsed chromosomes. **(c)** At late pachytene, the processing of recombination intermediates is complete and few high-intensity foci can be observed. The linear dotty signal has lost most of its intensity. **(d)** At diplotene, only foci can be observed and there is no trace of the linear dotty signal previously observed. Maximum projection is shown. Pattern of HEI10 was consistent in all cells analysed (n=141) in more than 10 independent experiments. Scale bar, 5 μm.

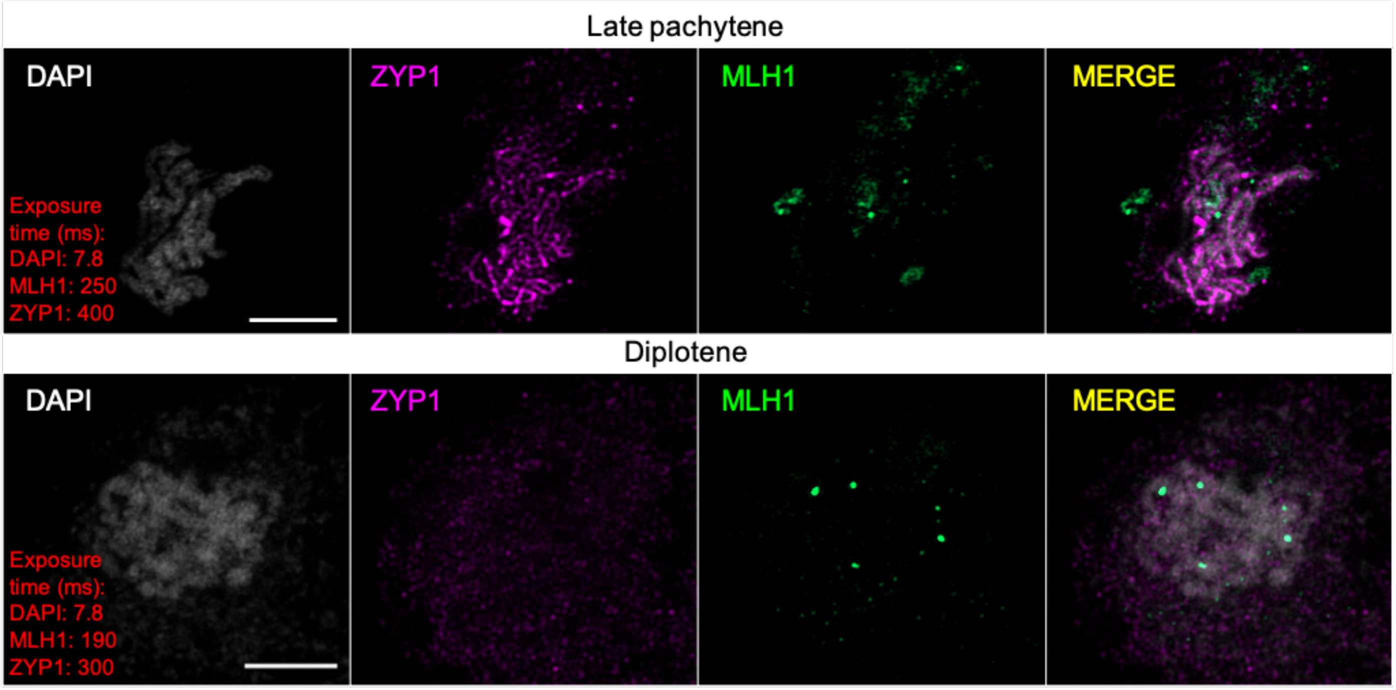

Supplementary Fig. 2: Dynamics of MLH1 during early prophase I in *R. breviuscula*. Note that MHL1 signals were never seen in early pachytene when ZYP1 signals were still present. MLH1 foci were only seen during diplotene and diakinesis when ZYP1 signals disappeared, representing the disassembly of the SC. Maximum projection is shown. DNA was counterstained with DAPI. Signal of MLH1 at late pachytene was extremely rare and reported in only this example during a single experiment. Scale bar, 5 µm.

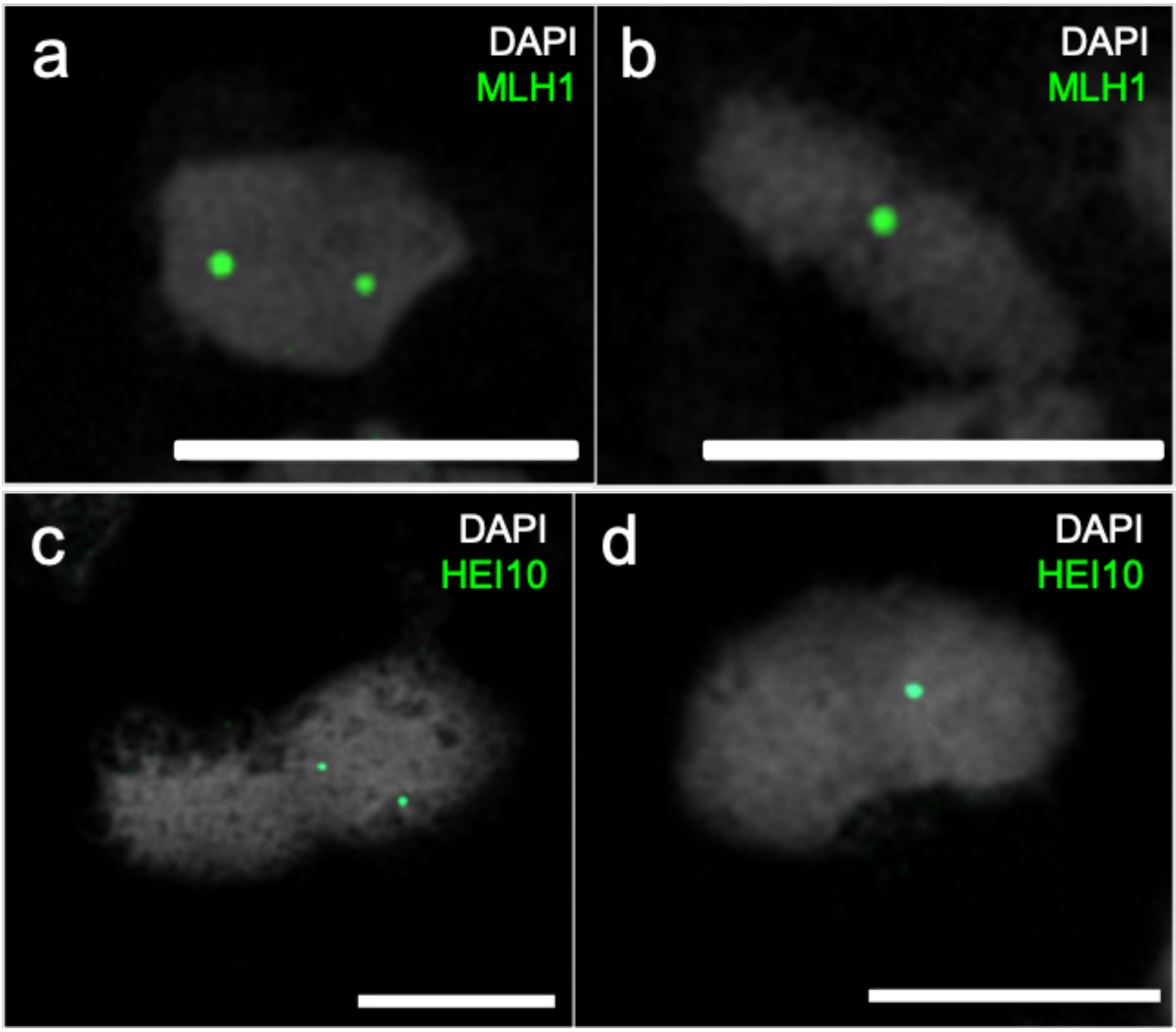

Supplementary Fig. 3: Examples of ring bivalents with two foci (a and c) and rod bivalents with one focus (b and d) of MLH1 and HEI10 at diakinesis in *R. breviuscula*. Maximum projection is shown. DNA was counterstained with DAPI. For Positioning of MLH1 foci on bivalents was consistent in all the cells at diakinesis (n=33) in five independent experiments. Positioning of HEI10 foci on bivalents was consistent in all the cells at diakinesis (n=17) in five independent experiments. Scale bars, 5 µm.

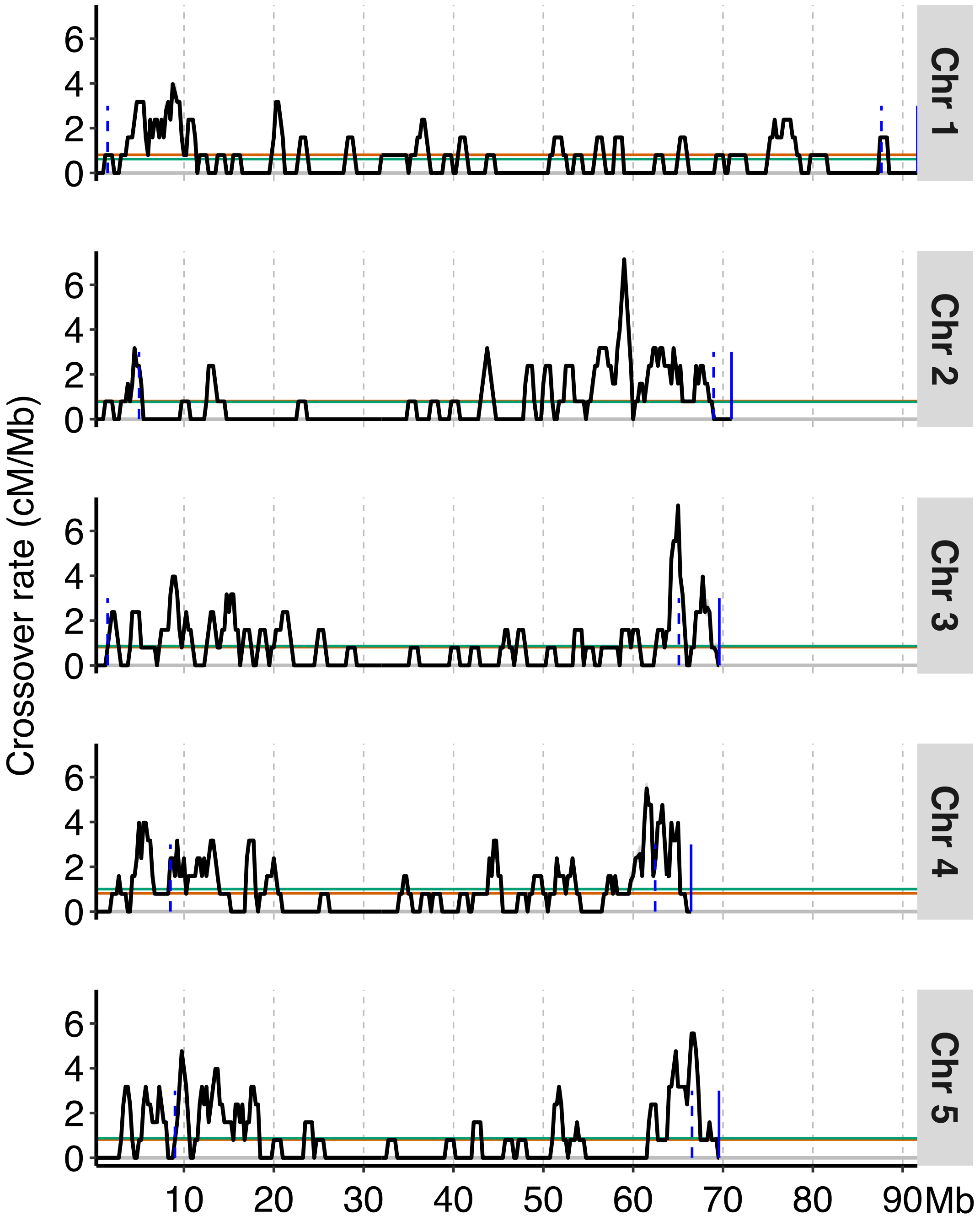

Supplementary Figure 4: Recombination landscape in the F1 recombinant offspring of *R. breviuscula*. Recombination landscape of the five chromosomes in *R. breviuscula* by computing 378 COs from 63 F1 offspring individuals. Black line displays the CO rate. Blue solid vertical lines indicate chromosomal ends. Orange horizontal line: genome-wide mean CO rate. Green horizontal line: chromosome-wide mean CO rate.

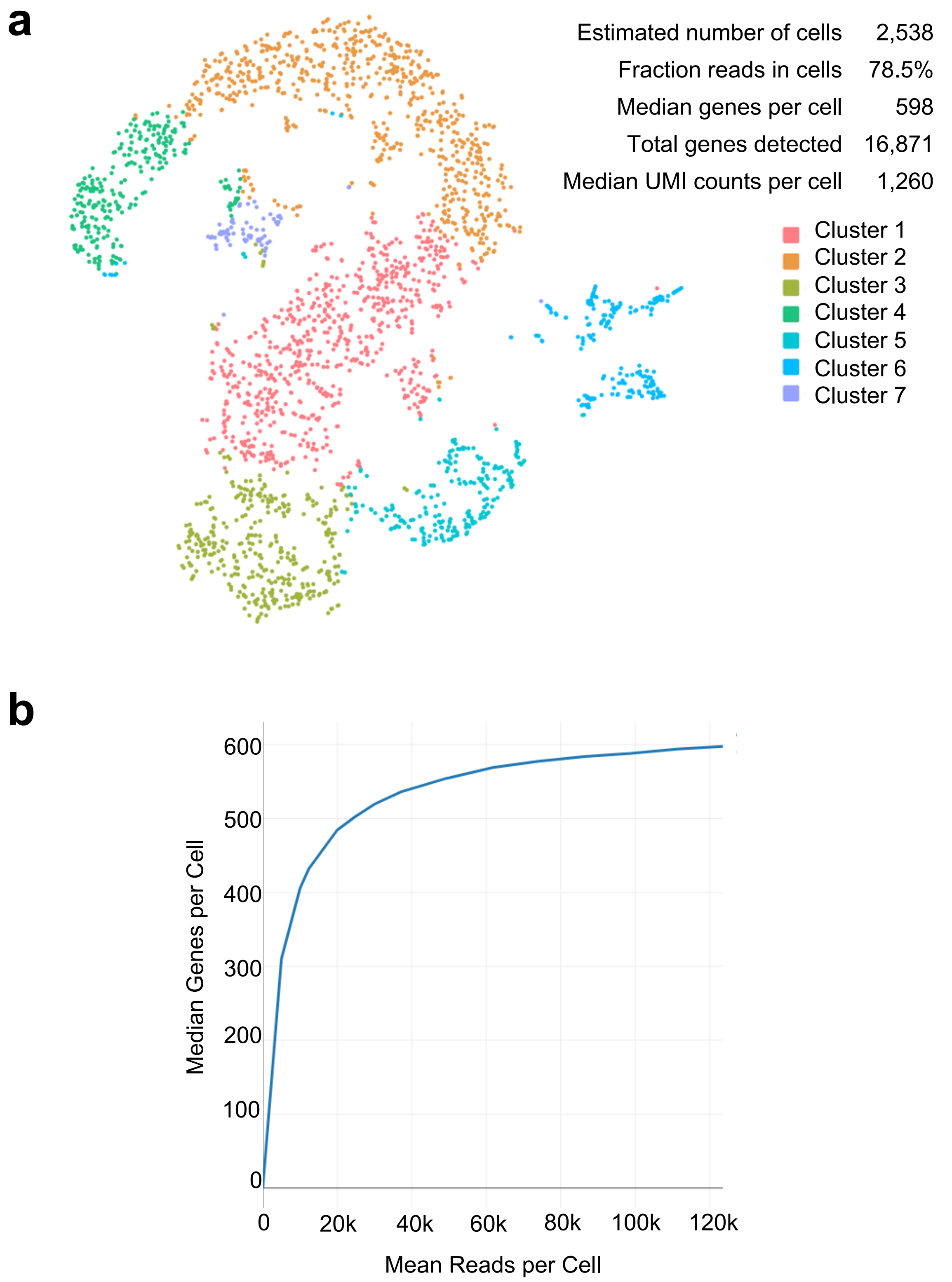

Supplementary Fig. 5: Cellranger clustering analysis of the 10X Genomics scRNA-seq of 2,528 pollen nuclei from *R. breviuscula*. (a) Clustering analysis identified 2,538 nuclei grouped in seven clusters composed of vegetative and generative pollen nuclei. **(b)** Curve showing the cumulative median number of genes per pollen nucleus as read coverage increase. Note the low median value of genes detected.

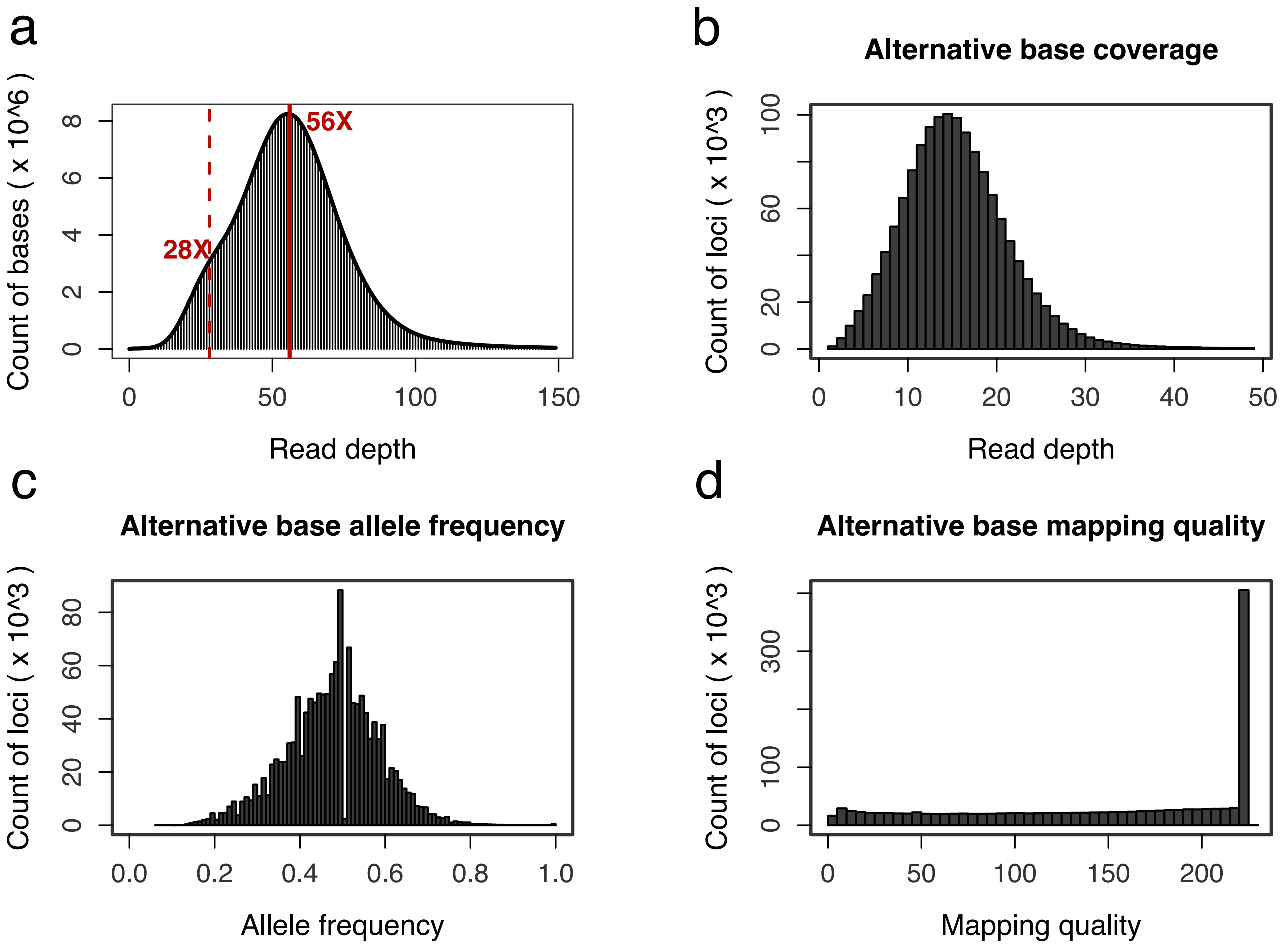

Supplementary Fig. 6: Selection of genotyping markers on the reference genome. (a) Distribution of read depth of Illumina reads mapping to haplotype 1 of the *R. breviuscula* phased genome. **(b–d)** Characteristics of alternative bases of SNPs that were called from the alignment mentioned in **(a)**. Genotyping markers on the reference were selected according to the distributions of coverage **(b)**, allele frequency **(c)**, and the mapping quality **(d)** of alternative bases. Specifically, an alternative base at a SNP position that met the requirements "5 ≤ alternative base coverage ≤ 30, 0.4 ≤ allele frequency ≤ 0.6, mapping quality > 50" was an allelic SNP, i.e., a genotyping marker.

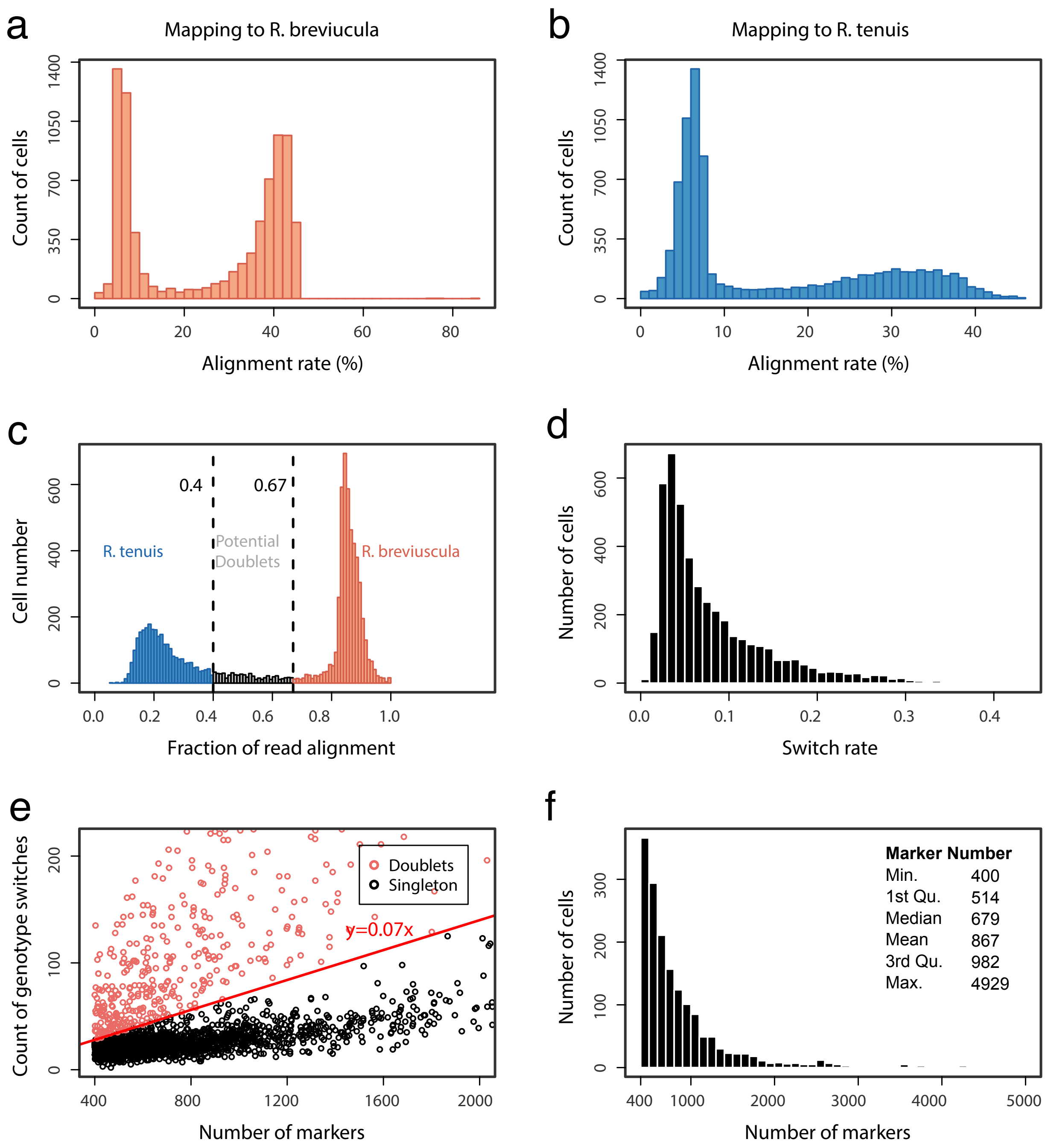

Supplementary Fig. 7: Pre-processing of scRNA-seq by separating *R. breviuscula* from *R. tenuis* cells and removing doublets. The scRNA-seq library was prepared with mixed pollen nuclei of *R. breviuscula* and *R. tenuis* for multiplexing purposes, thus it was necessary to discriminate the single-cell data between the two species at first. **(a–b)** Distribution of alignment rates of each read to *R. breviuscula* **(a)** and *R. tenuis* **(b)**. **(c)** Distribution of the fraction of reads from each cell aligning to *R. breviuscula* over the read alignments to both species, i.e., for a certain cell, fraction = number of reads mapped to *R. breviuscula* / (number of reads mapped to *R. breviuscula* + number of reads mapped to *R. tenuis*). Cells with an alignment fraction over 0.67 are potentially from *R. breviuscula*. Those with a fraction below 0.4 are potentially from *R. tenuis*. The remaining cells are doublets. **(d)** Distribution of switch rate across *R. breviuscula* pollens. The switch rate of a certain cell was calculated as the frequency of genotype switches between two consecutive markers over the total number of markers in this cell. **(e)** Identification of doublets by switch rates. Cells with a switch rate over 0.07 were considered doublets. **(f)** Number of markers across *R. breviuscula* pollen cells with a high number (≥400) of markers and no doublets.

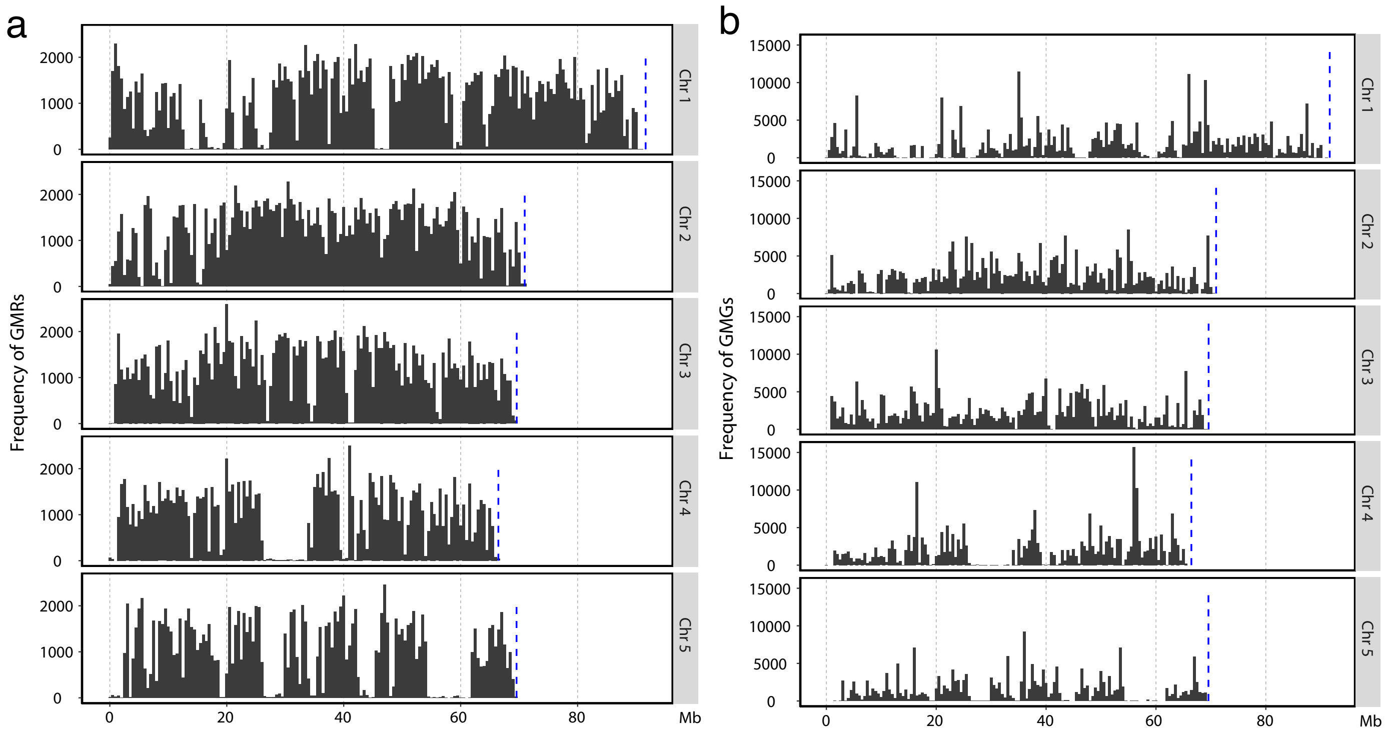

Supplementary Fig. 8: Marker distribution on the reference and across all viable pollen nuclei. (a) Frequency of genotyping markers defined along each chromosome on reference rhyBreHap1. Blue dashed lines show the end of each chromosome. GMR, genotype markers on reference genome. **(b)** Frequency of all markers across viable pollen nuclei that were used for CO detection. GMG, genotype markers on gametes.

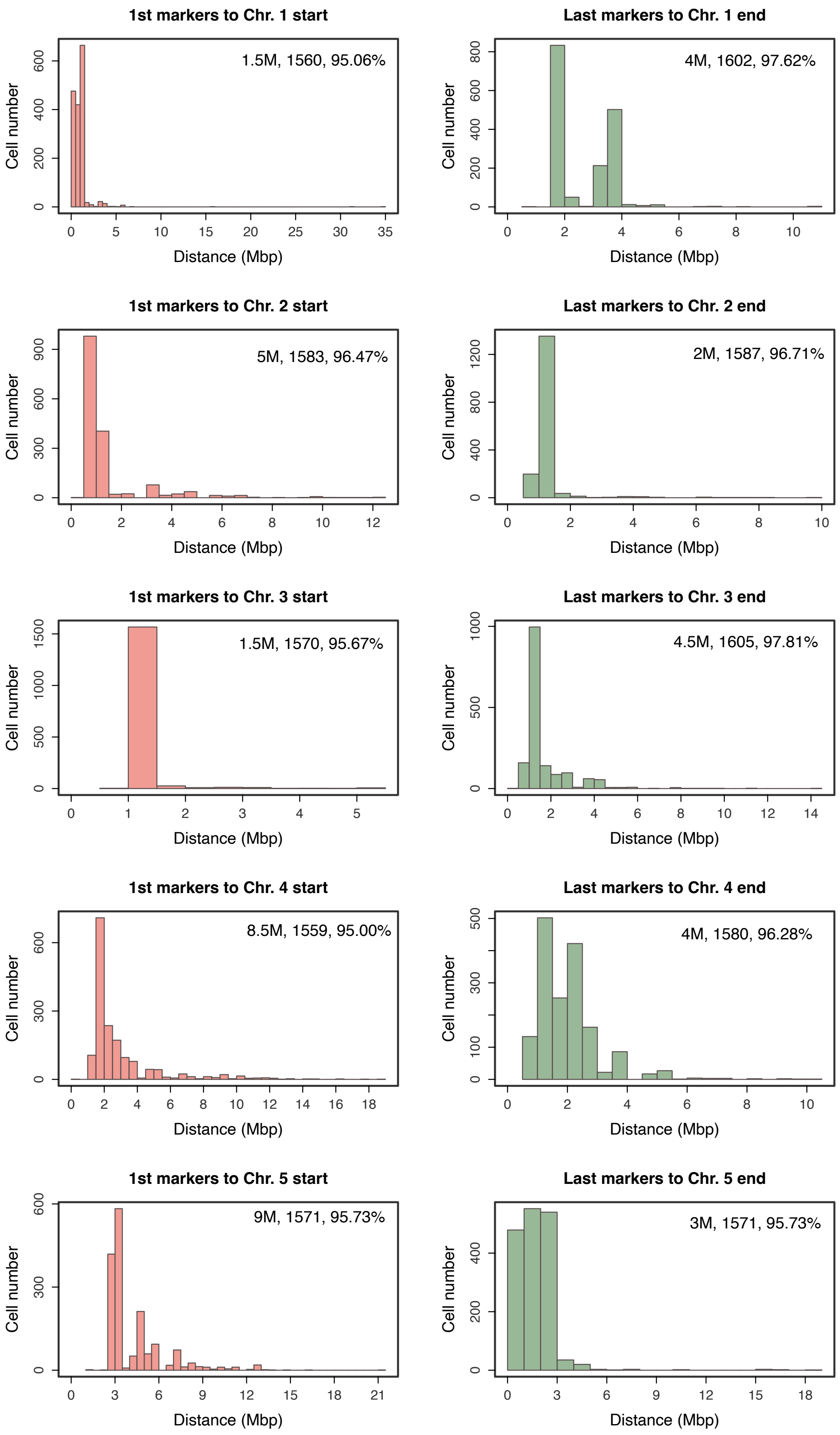

Supplementary Fig. 9: Distance distribution of the first markers to the chromosome start and the last markers to the chromosome ends across all viable pollen nuclei. If the regions covered by the first and last markers can be found in at least 95% of pollen nuclei, they are defined as confident start and end of the recombination landscape. The number on each plot indicates the distance of the confident regions to the chromosomal ends, the number of pollen nuclei covered, and the percentage of covered pollen nuclei.

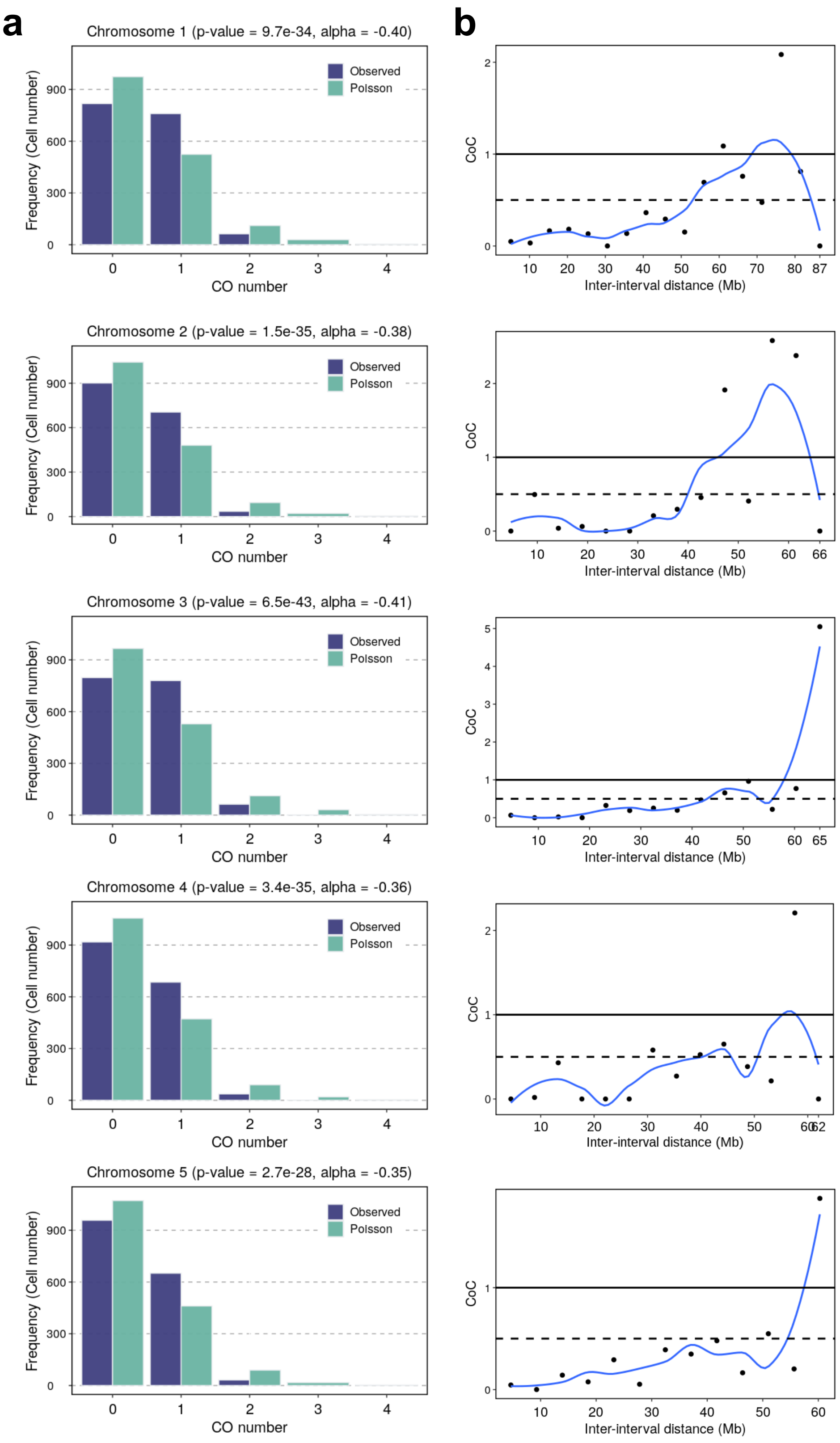

Supplementary Fig. 10: CO interference on each chromosome. (a) Comparison of observed CO number and expected CO number under the assumption of no interference on each chromosome. Chi-square values were first computed based on the chi-square goodness-of-fit test with a Poisson distribution. The *p*-value was computed based on a chi-square distribution with the above Chi-square value and degree of freedom. The alpha value was derived from the dispersion test. **(b)** CoC curve for each chromosome in the pollen nuclei (*n* = 1,641). Chromosome 1 was divided into 18 intervals and chromosomes 2–5 were divided into 15 intervals. Random sampling was performed on the CO intervals to calculate the mean CoC of each pair of intervals.

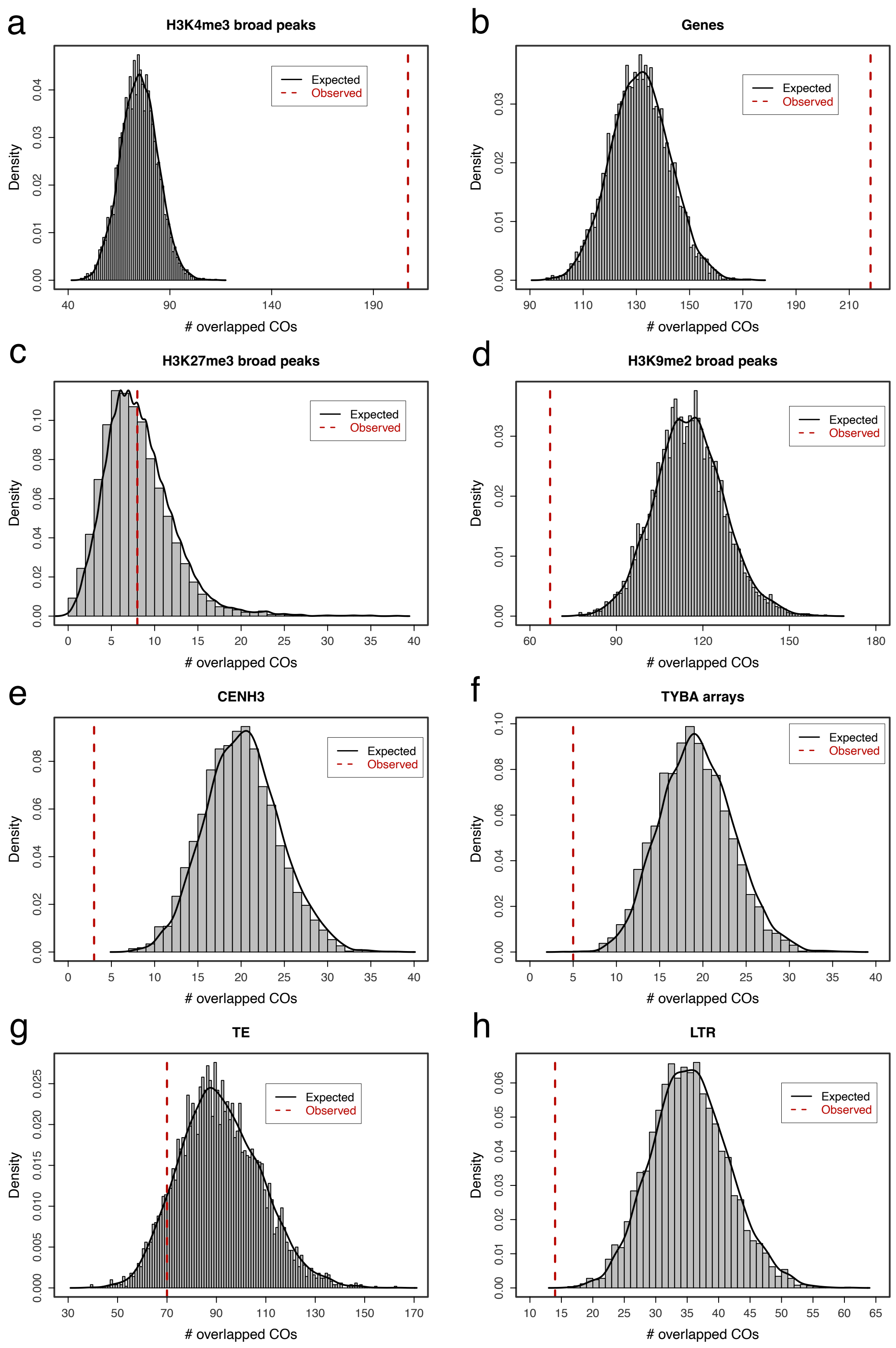

Supplementary Fig. 11: Comparison of numbers of COs overlapped with (epi)genetic features to random simulations. Observed overlapped CO number is displayed with red dashed vertical lines. Histograms show the distributions of overlapped CO numbers with H3K4me3 **(a)**, genes **(b)**, H3K27me3 **(c)**, H3K9me2 **(d)**, CENH3 **(e)**, *Tyba* arrays **(f)**, TEs **(g)**, and LTRs **(h)** in 5,000 simulations of randomly assigned COs.

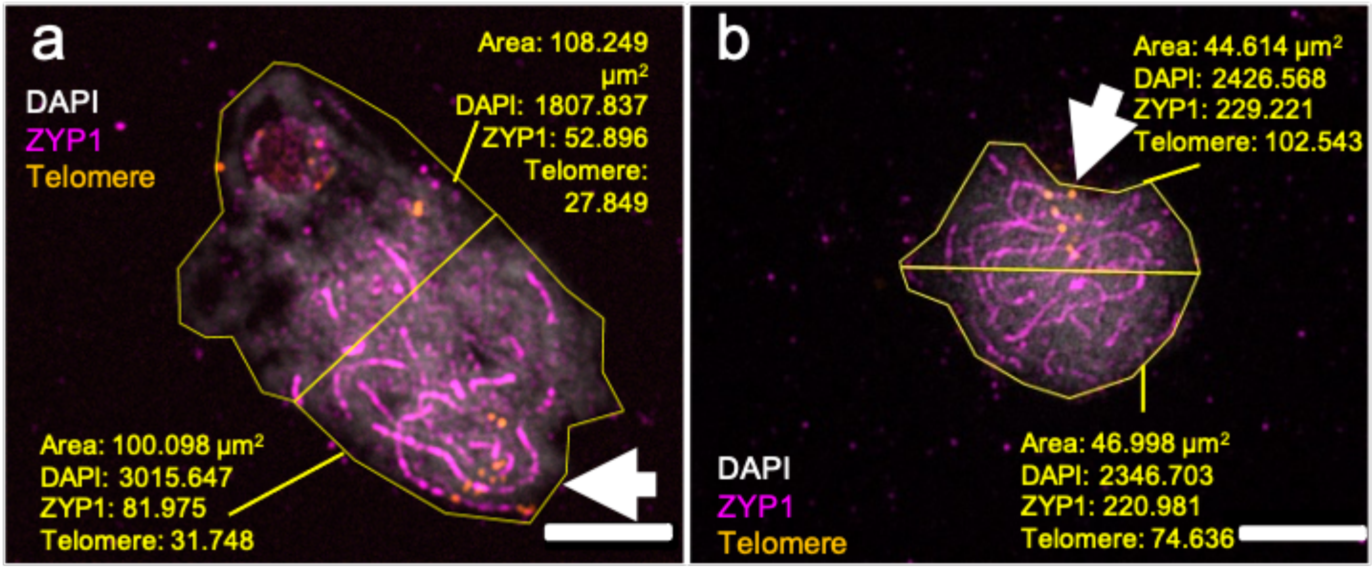

Supplementary Figure 12: Comparison of ZYP1 signal intensity between nuclear region adjacent to the telomere bouquet and region opposite to it in two examples. (a) At early zygotene, most of the cell is not yet involved in synapsis. We divided the cell in two areas of similar size and calculated the intensity ZYP1 signal in each, as an estimation of how much of the cell is synapsed. The region adjacent to the telomere *bouquet* (white arrow) displays a higher mean intensity of ZYP1 compared to the opposite region, suggesting that regions adjacent to telomeres are synapsed with priority. **(b)** Following the same concept, at late zygotene, most of the cell is actually involved in synapsis, however a small region is still unsynapsed and indeed displays a lower mean intensity of ZYP1 signal compared to the region adjacent to the telomere bouquet (white arrow). ZYP1 relative intensity was calculated in these 2 examples (n=2), but its behaviour is consistent in all our immuno-fish observations (see **Fig. 5**). Maximum projection is shown. Scale bars, 5 μm.

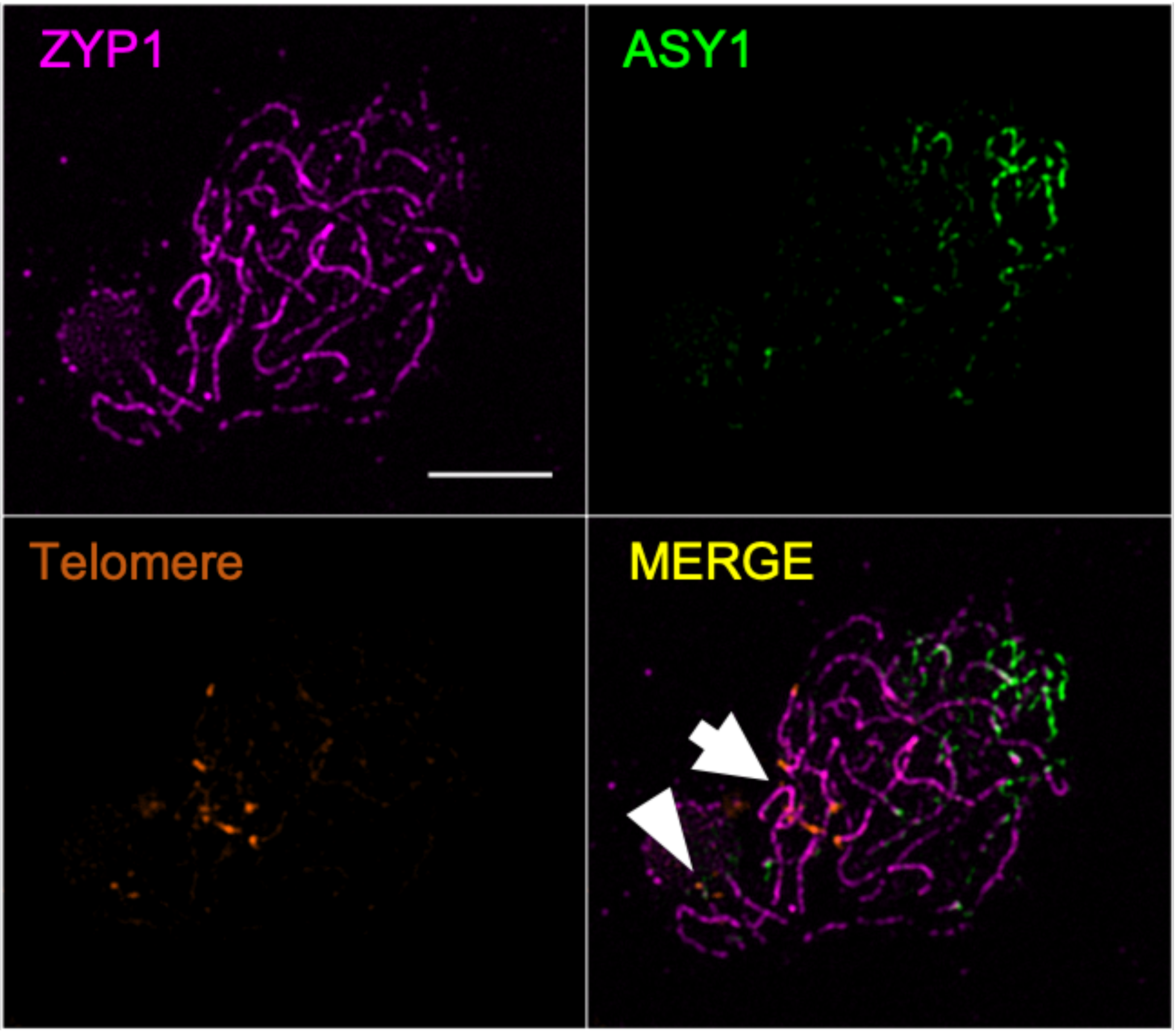

Supplementary Fig. 13: Immunolocalisation of ZYP1, ASY1 and telomere-FISH. In late zygotene, ASY1 represents unsynapsed chromosomes not yet reached by ZYP1, while ZYP1 occupies the rest of the chromosomal length loaded with the SC. Telomeres are still clustered in the *bouquet* (white arrow) or at the nucleolus (white arrowhead). Behaviour of ASY1+ZYP1+telomeres was consistent in all immuno-FISH cells (n=8) in 3 independent experiments. Scale bar, 5 μm.

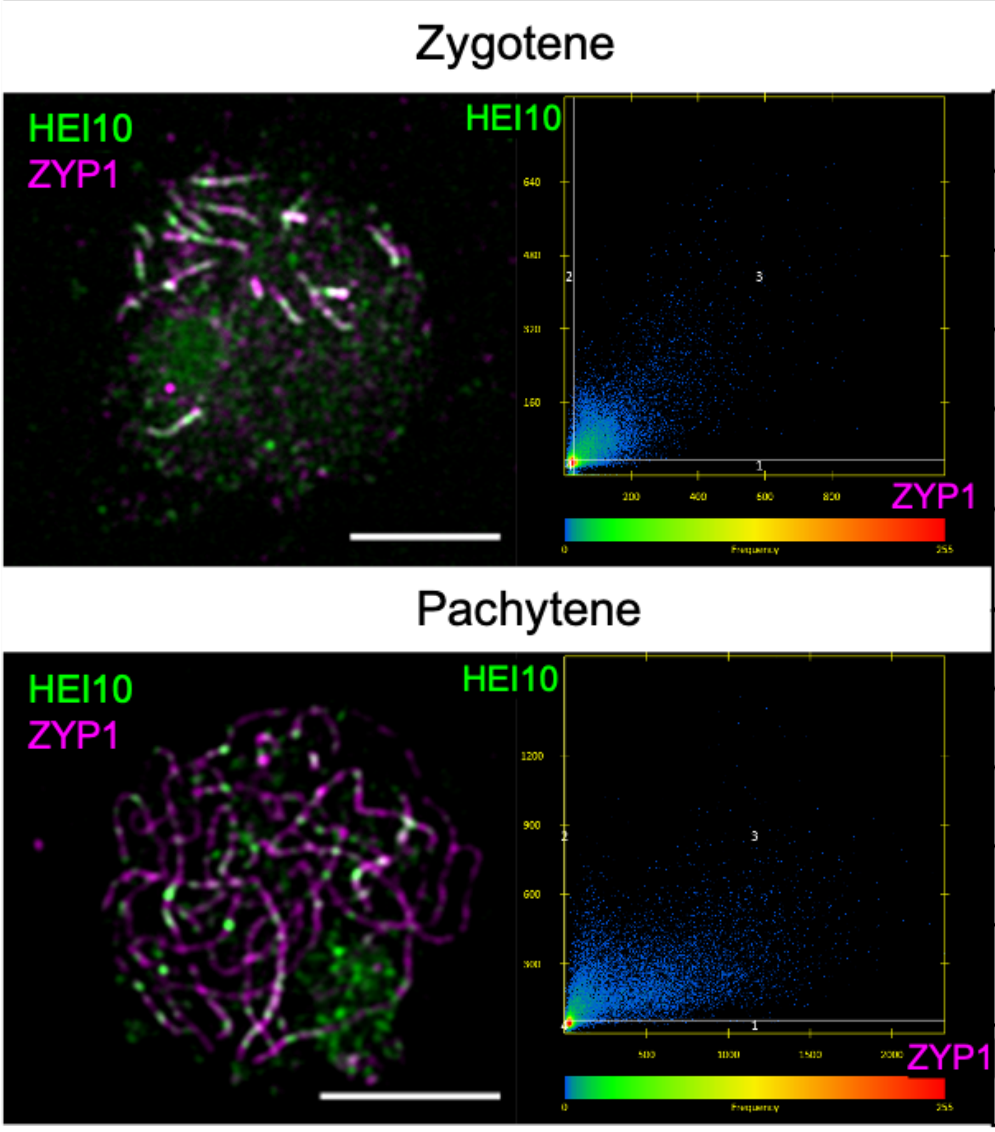

Pearson Coeff.	0.68437
Manders Coeff.	0.879
Coloc. Coeff. ZYP1	0.72302
Coloc. Coeff. HEI10	0.768
Weighted Coloc. Coeff. ZYP1	0.8609
Weighted Coloc. Coeff. HEI10	0.87287
Pearson Coeff.	0.56351
Manders Coeff.	0.79553
Coloc. Coeff. ZYP1	0.57618
Coloc. Coeff. HEI10	1
Weighted Coloc. Coeff. ZYP1	0.92247
Weighted Coloc. Coeff. HEI10	1

Supplementary Fig. 14: Colocalization analysis of processed images of meiocytes at zygotene (top) and pachytene (bottom). The analysis shows a positive and non-random colocalization between HEI10 and ZYP1 signals. Colocalization coefficient was calculated in these two examples, but the behaviour of HEI10 is consistent in all our cells from zygotene to late pachytene (n=61) in 10 independent experiments. Images were processed with ZEN software (Zeiss), utilizing the colocalization function. Intensity threshold was automatically selected by Costes significance algorithm. Scale bars, 5 μm.
