# Supplementary material for: Meiotic recombination dynamics in plants with repeat-based holocentromeres shed light on the primary drivers of crossover patterning
Source: Nat Plants. 2024 Feb 9;10(3):423–38. doi: 10.1038/s41477-024-01625-y (PMC10954556; doi:10.1038/s41477-024-01625-y)
Supplement: Supplementary file 2 — Reporting Summary [file 41477_2024_1625_MOESM2_ESM.pdf]

nature portfolio

Corresponding author(s): André Marques

Last updated by author(s): Jan 3, 2024

Reporting Summary

Nature Portfolio wishes to improve the reproducibility of the work that we publish. This form provides structure for consistency and transparency in reporting. For further information on Nature Portfolio policies, see our Editorial Policies and the Editorial Policy Checklist.

Statistics

For all statistical analyses, confirm that the following items are present in the figure legend, table legend, main text, or Methods section.

n/a	Confirmed	
☐	☒	The exact sample size (*n*) for each experimental group/condition, given as a discrete number and unit of measurement
☐	☒	A statement on whether measurements were taken from distinct samples or whether the same sample was measured repeatedly
☐	☒	The statistical test(s) used AND whether they are one- or two-sided<br>*Only common tests should be described solely by name; describe more complex techniques in the Methods section.*
☒	☐	A description of all covariates tested
☐	☒	A description of any assumptions or corrections, such as tests of normality and adjustment for multiple comparisons
☐	☒	A full description of the statistical parameters including central tendency (e.g. means) or other basic estimates (e.g. regression coefficient) AND variation (e.g. standard deviation) or associated estimates of uncertainty (e.g. confidence intervals)
☐	☒	For null hypothesis testing, the test statistic (e.g. *F*, *t*, *r*) with confidence intervals, effect sizes, degrees of freedom and *P* value noted<br>*Give P values as exact values whenever suitable.*
☒	☐	For Bayesian analysis, information on the choice of priors and Markov chain Monte Carlo settings
☒	☐	For hierarchical and complex designs, identification of the appropriate level for tests and full reporting of outcomes
☐	☒	Estimates of effect sizes (e.g. Cohen's *d*, Pearson's *r*), indicating how they were calculated

Our web collection on statistics for biologists contains articles on many of the points above.

Software and code

Policy information about availability of computer code

Data collection

Sorting of pollen nuclei was performed by a BD FACSAria Fusion sorter (BD Biosciences). The Epifluorescence microscope Zeiss Axio Imager Z2 with Apotome system for optical sectioning and Leica Microsystems Thunder Imager dMi8 with Computational Clearing. were used for acquiring microscopic images.

Data analysis

Custom code:
CO detection pipeline by scRNA-seq (https://github.com/Raina-M/detectCO_by_scRNAseq)

Other available open source tools used in this study:
AHRD pipeline (v3.3.3; https://github.com/groupschoof/AHRD)
Augustus (v3.3.3)
Deeptools (v3.5.1)
Bcctools (v0.1.1)
Bcftools (v1.9)
Bedtools (v2.29.0)
Bismark (v0.23.0)
bowtie2 (v2.4.4)
BUSCO (v5.1.2)
Cuffcompare (v2.1.1)
Cutadapt (v4.7)
DIAMOND (v2.0.5)
EVidenceModeller (v1.1.1)

GeneMark (v4.35)
GenomeThreader (v1.7.1)
GMAP (2018-07-04)
HiFiasm (0.19.5-r592)
Hisat2 (v2.1.0)
KaKs_Calculator (v3)
karyoploteR (v3.18)
MACS3 (v3.0.0)
MADpattern (v1.1)
minimap2 (v2.26)
PASA (v2.4.1)
ParaAT (v2)
pyGenomeTracks (v3.8)
RepeatExplorer2 (v2.3.7)
RTIGER (v1.99.0)
SALSA2 (v2.3)
Samtools (v1.9)
SHOREmap (v3.6)
STAR (v2.7.8a)
StringTie (v2.1.5)
SyRI (v1.5)
TIGER (v1.0)
TransDecoder (v5.5.0)
UMIcollapse (v1.0.0)

For manuscripts utilizing custom algorithms or software that are central to the research but not yet described in published literature, software must be made available to editors and reviewers. We strongly encourage code deposition in a community repository (e.g. GitHub). See the Nature Portfolio guidelines for submitting code & software for further information.

Data

Policy information about availability of data

All manuscripts must include a data availability statement. This statement should provide the following information, where applicable:

- Accession codes, unique identifiers, or web links for publicly available datasets
- A description of any restrictions on data availability
- For clinical datasets or third party data, please ensure that the statement adheres to our policy

All sequencing data used in this study have been deposited at NCBI under the BioProject ID PRJNA1059790 and are publicly available as of the date of publication. The reference genomes, sequencing data, annotations and all tracks presented in this work are made available for download at DRYAD: https://datadryad.org/stash/share/EvB3PRNVph5IiTkOM3jTZddgmS45cJhQYq2v3LI5InE. The REXdb database Viridiplantae v3.0 [http://repeatexplorer.org/?page_id=918] is publicly available. All other data needed to evaluate the conclusions in the paper are provided in the paper and/or the supplemental information.

Research involving human participants, their data, or biological material

Policy information about studies with human participants or human data. See also policy information about sex, gender (identity/presentation), and sexual orientation and race, ethnicity and racism.

Reporting on sex and gender	n/a
Reporting on race, ethnicity, or other socially relevant groupings	n/a
Population characteristics	n/a
Recruitment	n/a
Ethics oversight	n/a

Note that full information on the approval of the study protocol must also be provided in the manuscript.

Field-specific reporting

Please select the one below that is the best fit for your research. If you are not sure, read the appropriate sections before making your selection.

☒ Life sciences ☐ Behavioural & social sciences ☐ Ecological, evolutionary & environmental sciences

For a reference copy of the document with all sections, see nature.com/documents/nr-reporting-summary-flat.pdf

Life sciences study design

All studies must disclose on these points even when the disclosure is negative.

Sample size	Sample-size calculation was performed based on assessment of the literature in the field, our own experience from previous studies and requirement for corresponding protocols. For Immunocytochemistry analyis sample size was based on the number of cells obtained. For single-cell sequencing, sample size was determined based on the number of cells that can be typically obtained using 10X scRNAseq libraries. For F1 samples, the sample size was based on the maximum number of seeds obtained by selfing. The sample size used for all experiments provided sufficient resolving power.
Data exclusions	10X Genomics scRNA library was initially performed by combining pollen grains from R. breviuscula and R. tenuis, for multiplex purposes. However, all the pollen data from R. tenuis was excluded from the analysis, as this will be used in separate manuscript.
Replication	Replication was mainly used for our ChIP experiments, which were all done in two replicates. We confirm that all replicates showed similar results, confirming the reproducibility of our analysis. Furthermore, cytological analyses were performed several times in different days and with different fixations using the same set of antibodies. We confirm that our cytological data is very reproducible. Individual F1 plants WGS and scRNA sequencing approaches were not replicated, since each individual pollen nuclei or F1 plants already represent biological replicates of recombination events.
Randomization	A randomization is not relevant for this study because no genotype or treatment were compared with each other. However, the tissues for cytogenetic and ChIPseq experiments were randomly collected from different plant individuals grown under the same condition in a greenhouse. Only for scRNAseq, we used the pollen collected from a single R. breviuscula plant as this needs to match the same heterozygous polymorphisms of the reference mother plant in order to detect COs using our method.
Blinding	All the experiments were performed without prior knowledge of the final outcome, and therefore blinding was not applied.

Reporting for specific materials, systems and methods

We require information from authors about some types of materials, experimental systems and methods used in many studies. Here, indicate whether each material, system or method listed is relevant to your study. If you are not sure if a list item applies to your research, read the appropriate section before selecting a response.

Materials & experimental systems

n/a	Involved in the study	
☐	☒	Antibodies
☒	☐	Eukaryotic cell lines
☒	☐	Palaeontology and archaeology
☒	☐	Animals and other organisms
☒	☐	Clinical data
☒	☐	Dual use research of concern
☐	☒	Plants

Methods

n/a	Involved in the study	
☐	☒	ChIP-seq
☒	☐	Flow cytometry
☒	☐	MRI-based neuroimaging

Antibodies

Antibodies used

Customized Rhynchospora-specific antibodies generated in this study:
1. anti-REC8 was a combination of two antibodies raised in rabbits against the Rhynchospora-specific REC8-peptides C-EEPYGEIQISKGPNM and C-YNPDDSVERMRDDPG (gene ID: RBREV_HAP1.r01.Chr4_h1G00395720.1) and affinity-purified (Eurogentec).
2. anti-HEI10 was a combination of two antibodies raised in rabbits against the Rhynchospora-specific HEI10-peptides C-NRPNQSRARTNMFQL and C-PVRQRNNKSMVSGGP (gene ID: RBREV_HAP1.r01.Chr4_h1G00387160.1) and affinity-purified (Eurogentec).

Previously designed antibodies:
3. anti-AtASY1 raised in rabbits (inventory code PAK006) (Armstrong et al. 2002)
4. anti-AtMLH1 raised in rabbits (PAK017) (Chelysheva et al. 2010)
5. anti-RhynchosporaCENH3 raised in rabbits (Marques et al. 2015).
6. anti-ZYP1 was raised in chickens against the peptide EGSLNPYADDPYAFD of the C-terminal end of AtZYP1a/b (gene ID: At1g22260/At1g22275) and affinity-purified (Eurogentec) (PAK048).
Each primary antibody above was diluted 1:200 in blocking solution.

Commercially available antibodies:
7. anti-H3K4me3 (rabbit polyclonal to Histone H3 tri-methyl K4; Abcam, UK, cat. no. ab8580, dilution 1:300)
8. anti-H3K9me2 (mouse monoclonal to Histone H3 di-methyl K9, Abcam, UK, cat. no. ab1220, clone no. mAbcam 1220, dilution 1:200)
9. anti-H3K27me3 (mouse monoclonal to Histone H3 tri-methyl K27, Abcam, UK, cat. no. ab6002, clone no. mAbcam 6002, dilution 1:200)
10. anti-IgG control (recombinant rabbit IgG, monoclonal Abcam, UK, cat. no. ab172730, clone no. EPR25A, dilution 1:300).

Commercially available secondary antibodies:
11. Abberior STAR ORANGE (dilution 1:250, Abberior, DE, goat anti-rabbit IgG, cat. no. STORANGE-1002-500UG or goat anti-chicken IgY, cat. no. STORANGE-1005-500UG)
12. Abberior STAR RED (dilution 1:250, Abberior, DE, goat anti-rabbit IgG, cat. no. STRED-1002-500UG or goat anti-chicken IgY, cat. no. STRED-1005-500UG)

Validation

Previously validated antibodies;
1. Anti-AtASY1 (inventory code PAK006) was validated in Armstrong et al. (2002).
2. Anti-AtMLH1 (PAK017) was validated in Chelysheva et al. (2010)
3. Anti-RhynchosporaCENH3 was validated in Marques et al. (2015, 2016)

Validation by commercial providers:
4. anti-H3K4me3 (cat. no. ab8580, https://www.abcam.com/products/primary-antibodies/histone-h3-tri-methyl-k4-antibody-chip-grade-ab8580.html)
5. anti-H3K9me2 (cat. no. ab1220, https://www.abcam.com/products/primary-antibodies/histone-h3-di-methyl-k9-antibody-mabcam-1220-chip-grade-ab1220.html)
6. anti-H3K27me3 (cat. no. ab6002, https://www.abcam.com/products/primary-antibodies/histone-h3-tri-methyl-k27-antibody-mabcam-6002-chip-grade-ab6002.html)
7. anti-IgG (cat. no. ab172730, https://www.abcam.com/products/primary-antibodies/rabbit-igg-monoclonal-epr25a-isotype-control-ab172730.html)

Newly validated antibodies:
4. Anti-ZYP1 antibody was generated by the company EUROGENTEC and validated by peptide ELISA tests. Further validation was demonstrated in A. thaliana by showing positive staining of ZYP1 in wildtype, but not in zyp1 mutants (data not shown). Remarkably, this peptide region showed 100% similarity with the Rhynchospora ZYP1 C-terminal (gene ID: RBREV_HAP1.r01.Chr2_h1G00222020.1).
Rhynchospora-specific REC8 and HEI10 antibodies were generated by the company EUROGENTEC and validated by peptide ELISA test. Further validation was obtained by the presence of specific signals on western blots (data not shown). Both ELISA and western blot information are available upon request. Furthermore, the observed indirect immuno-signals of anti-ZYP1, anti-HEI10 and anti-REC8 are compatible with data previously reported in the published literature for other species.

Plants

Seed stocks

Individual plants from R. breviuscula were previously obtained by Hofstatter et al. 2022 and kept under cultivation at growth chambers at the Max Planck Institute for Plant Breeding Research in Cologne, Germany. A F1 progeny was obtained by selfing the original heterozygous mother plant used in Hofstatter et al. 2022. Additionally, all cytology and pollen were collected from the same heterozygous mother plant genotype.

Novel plant genotypes

Describe the methods by which all novel plant genotypes were produced. This includes those generated by transgenic approaches, gene editing, chemical/radiation-based mutagenesis and hybridization. For transgenic lines, describe the transformation method, the number of independent lines analyzed and the generation upon which experiments were performed. For gene-edited lines, describe the editor used, the endogenous sequence targeted for editing, the targeting guide RNA sequence (if applicable) and how the editor was applied.

Authentication

Describe any authentication procedures for each seed stock used or novel genotype generated. Describe any experiments used to assess the effect of a mutation and, where applicable, how potential secondary effects (e.g. second site T-DNA insertions, mosiacism, off-target gene editing) were examined.

ChIP-seq

Data deposition

☒ Confirm that both raw and final processed data have been deposited in a public database such as GEO.

☒ Confirm that you have deposited or provided access to graph files (e.g. BED files) for the called peaks.

Data access links
May remain private before publication.

https://datadryad.org/stash/share/EvB3PRNVph5IiTkOM3jTZddgmS45cJhQYq2v3LI5InE

Files in database submission

Phased genome assemblies of two haplotypes of R. breviuscula

rhyBreHap1.fasta.gz

rhyBreHap2.fasta.gz

Annotation of phased genome assemblies

rhyBreHap1_functAnno.gff3.gz

rhyBreHap2_functAnno.gff3.gz

Single-cell RNA sequences from pollen nuclei by 10X Genomics

a4984_merged_R1.fastq.gz

a4984_merged_R1.fastq.gz

Please note that the scRNA-seq data were sequenced from mixed R. breviuscula and R. tenuis pollen nuclei. Therefore, you need to separate the pollen of two individuals before any further analysis. To do this, you can refer to the online methods of the above paper and our github docbumentation.

Whole-genome DNA short reads of 63 selfed F1 offspring by Illumina paired-end sequencing

F1_WGS.tar.gz

WGS DNA reads of all 63 F1 individuals are in this compressed folder. Sample IDs are 5445_A, 5445_B, 5445_C, 5445_D, 5445_E, 5621_A, 5621_B, 5621_C, 5621_E, 5621_F, 5621_G, 5621_H, 5621_I, 5621_J, 5621_K, 5621_L, 5621_M, 5621_N, 5621_O, 5621_P, 5621_Q, 5621_R, 5621_S, 5621_T, 5621_U, 5621_V, 5621_W, 5621_X, 5621_Y, 5844_A, 5844_B, 5844_C, 5844_D, 5844_E, 5844_F, 5844_G, 5844_H, 5844_I, 5844_J, 5844_K, 5844_L, 5844_M, 5844_N, 5844_O, 5844_P, 5844_Q, 5844_R, 5844_S, 5844_T, 5844_U, 5844_V, 5844_W, 5844_X, 5844_Y, 5844_Z, 5844_AA, 5844_AB, 5844_AC, 5844_AD, 5844_AE, 5844_AF, 5844_AG, 5844_AH. Each sample was sequenced under one single library, so you can merge the reads with the same sample names.

ChIPseq data

ChIPseq_raw_reads.tar.gz

5165_A CENH3 rep1 rabbit

5165_B CENH3 rep2 rabbit

5165_C H3K4me3 rep1 rabbit

5165_D H4K4me3 rep2 rabbit

5165_E H3K9me2 rep1 mouse

5165_F H3K9me2 rep2 mouse

5165_G Rabbit-IgG rep1 rabbit

5165_H Rabbit-IgG input rep2 rabbit

5165_I input chromatin rep1

5165_J input chromatin rep2

5165_M Mouse-IgG rep1 mouse

5165_N Mouse-IgG rep2 mouse

5165_O H3K27me3 rep1 rabbit

5165_P H3K27me3 rep2 rabbit

Each sample was sequenced under one single library, so you can merge the reads with the same sample names.

ChIP_peaks.zip

Methyl-seq data

Rhync_breviuscula_Methyl-seq.tar.gz

Each sample was sequenced under one single library, so you can merge the reads with the same sample names.

Complete CO detection pipeline by pollen scRNA-seq data can be found on this github page.

Genome browser session
(e.g. UCSC)

no longer applicable

Methodology

Replicates

ChIP experiments were performed in two technical replicates for each antibody used. All replicates agreed by showing similar enrichment results.

Sequencing depth

Each replicate was sequenced at an approx. 3.75 genome sequencing depth, i.e., 10 million of 150bp single-end reads. Alignment

Sequencing depth

rate >90% for all samples. CENH3 uniquely mapped reads >28% for both replicates. H3K4me3 uniquely mapped reads >45% for both replicates. H3K9me2 uniquely mapped reads >20% for both replicates. H3K27me3 uniquely mapped reads >37% for both replicates.

Antibodies

CENH3 ChIP-seq data were obtained from Hofstatter et al. (2022). Further ChIP experiments were performed for H3K4me3 (rabbit polyclonal to Histone H3 tri-methyl K4; Abcam ab8580), H3K9me2 (mouse monoclonal to Histone H3 di-methyl K9, Abcam ab1220), H3K27me3 (mouse monoclonal to Histone H3 tri-methyl K27, Abcam ab6002), and the IgG control (recombinant rabbit IgG, monoclonal Abcam ab172730) using the same protocol described by Hofstatter et al. (2022).

Peak calling parameters

MACS3 software was used for peak calling: macs3 callpeak -t ChIP.bam -c Control.bam --broad -g 380000000 --broad-cutoff 0.1

Data quality

Quality of peaks were checked by comparing the enrichment of immunoprecitated DNA in comparison to our controls, i.e., input chromatin and IgG, using deeptools. All peaks retained showed a fold enrichment above 5 and FDR less than 5%.

Software

bowtie2, MACS3, deeptools (bamCoverage, bamCompare, computeMatrix, plotHeatmap)
